# An Efficient Synthesis of Naphtho[2,3-*b*]furan-4,9-diones via Visible-Light-Mediated [3+2] Cycloaddition Reaction

**DOI:** 10.3390/molecules28124751

**Published:** 2023-06-13

**Authors:** Hongbo Tan, Zehui Qi, Yuanhui Yu, Xu Zhang, Yuheng Xiang, Jingwen Huang, Zhigang Xu, Dianyong Tang, Zhongzhu Chen, Bochu Wang

**Affiliations:** 1 National & Local Joint Engineering Research Center of Targeted and Innovative Therapeutics, Chongqing Engineering Laboratory of Targeted and Innovative Therapeutics, Chongqing Key Laboratory of Kinase Modulators as Innovative Medicine, Chongqing Collaborative Innovation Center of Targeted and Innovative Therapeutics, College of Pharmacy & IATTI, Chongqing University of Arts and Sciences, Chongqing 402160, China; 19122059273@163.com (Z.Q.); 18883085146@163.com (Y.Y.); 13637765110@163.com (X.Z.); 19946770877@163.com (Y.X.); hjw69328@163.com (J.H.); xzg@cqwu.com.cn (Z.X.); tangdy2008@163.com (D.T.); 18883138277@163.com (Z.C.); 2 Key Laboratory of Biorheological Science and Technology, Ministry of Education, College of Bioengineering, Chongqing University, Chongqing 400030, China; wangbc2000@126.com; 3 Chongqing Academy of Chinese Materia Medica, Chongqing 400065, China

**Keywords:** green chemistry, photochemistry, visible-light photocatalysis, [3+2] cycloaddition, naphtho[2,3-*b*]furan-4,9-diones, dihydronaphtho[2,3-*b*]furan-4,9-diones

## Abstract

Naphtho[2,3-*b*]furan-4,9-dione is an important privileged structural motif which is present in natural products, drugs, and drug candidates. Herein, visible-light-mediated [3+2] cycloaddition reaction for the synthesis of naphtho[2,3-*b*]furan-4,9-diones and dihydronaphtho[2,3-*b*]furan-4,9-diones has been developed. Under environmentally friendly conditions, a variety of title compounds were delivered in good yields. This new protocol shows excellent regioselectivity and remarkable functional group tolerance. This approach provides a powerful, green, efficient, and facile means to expand the structural diversity of naphtho[2,3-*b*]furan-4,9-diones and dihydronaph-tho[2,3-*b*]furan-4,9-diones as promising scaffolds for novel drug discovery.

## 1. Introduction

**Scheme 1:**
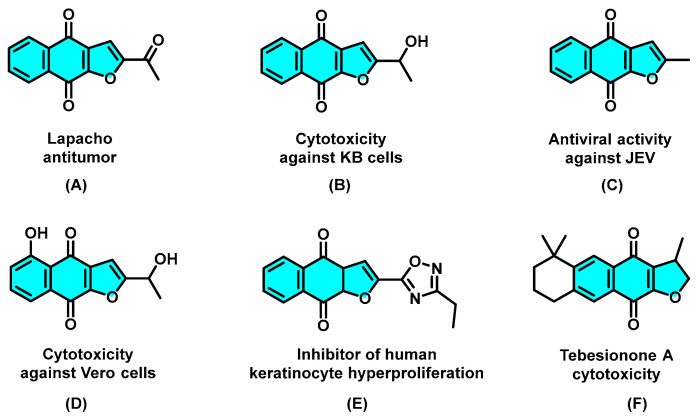
Representative biologically active naphtho[2,3-*b*]furan-4,9-diones.

**Scheme 2:**
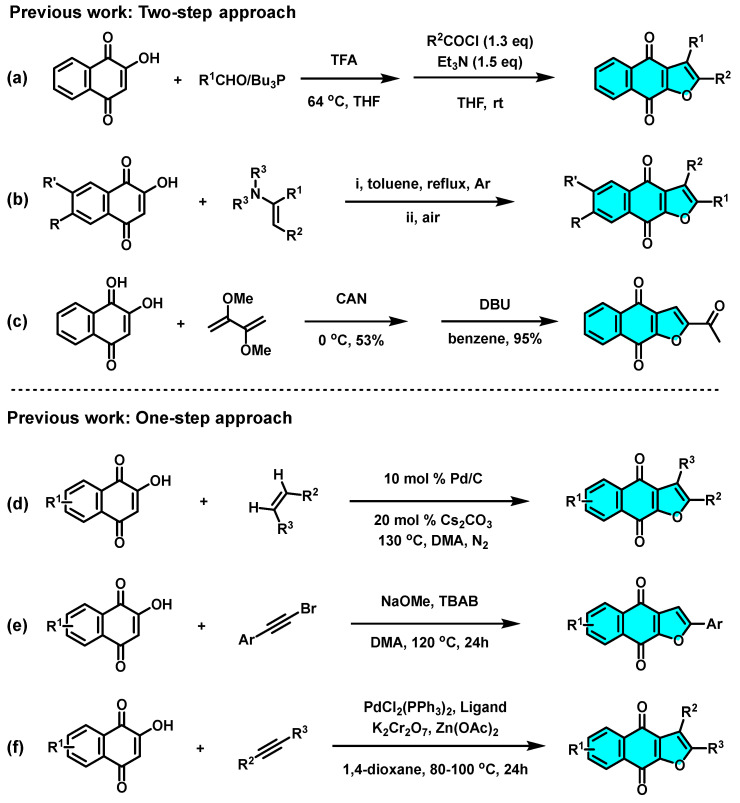
Synthetic approaches starting from 2-hydroxy-1,4-naphthoquinones: (**a**) multi-component reaction, (**b**) thermal cyclization with enamines, (**c**) CAN-mediated oxidative cycloaddition with enol ether, (**d**) transition-metal promoted thermal cyclization, (**e**) strong-base promoted thermal cyclization, (**f**) strong oxidantpromoted thermal cyclization.

As an important privileged structural motif, many naturally occurring naphtho[2,3-*b*]furan-4,9-diones and synthetic analogs have been widely found to exhibit versatile biological activities [1,2,3,4,5,6,7,8,9], in particular, antitumor [10], cytotoxic activity toward KB [11], antiviral activity against the Japanese encephalitis virus [12], and Vero cells [13], an inhibitor of human keratinocyte hyperproliferation [14], and other cytotoxic activities [15] (A−F, Scheme 1). Because of the importance of furonaphthoquinones to pharmaceutical research and drug discovery, considerable efforts have been focused on the synthetic approaches of naphtho[2,3-*b*]furan-4,9-dione ring system. In recent years, different methodologies for the synthesis of furonaphthoquinones have been reported (Scheme 2). Predominantly starting from 2-hydroxy-1,4-naphthoquinones, the two-step procedures, such as multi-component reaction (Scheme 2a) [16], thermal cyclization with enamines (Scheme 2b) [17], and CAN-mediated oxidative cycloaddition with enol ether (Scheme 2c), [18] and one-step cascade approaches, such as transition-metal (Scheme 2d) [19] or strong-base (Scheme 2e) [20] or strong oxidant (Scheme 2f) [21] promoted thermal cyclization methods have been developed. In addition, other multifarious methods, such as Friedel−Crafts acylation/oxidation [22] and bromine-mediated intramolecular cyclization [23], have also been developed. Although significant progress has been made in the synthesis of naphtho[2,3-*b*]furan-4,9-diones, novel green synthetic approaches with milder reaction conditions and enhanced reaction efficiency are still desirable.

**Scheme 3:**
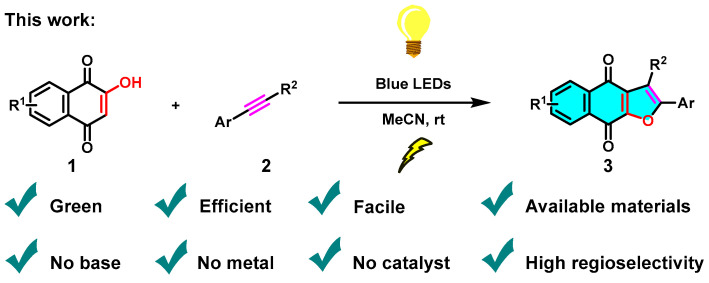
Visible-light-mediated green synthesis of naphtho[2,3-*b*]furan-4,9-diones.

To supplement our initial research in the synthesis of heterocyclic compounds by green MCR approaches and photochemical protocols [24,25,26,27], herein, we report a concise, efficient and green synthetic approach that affords naphtho[2,3-*b*]furan-4,9-diones via the visible-light-mediated [3+2] cycloaddition reaction of 2-hydroxy-1,4-naphthoquinones (**1**) and phenylacetylenes (**2**) under irradiation of blue LEDs (460 nm) in the absence of any bases, metals, ligands, or other catalysts (Scheme 3).

## 2. Results and Discussion

In the pilot experiment, 2-hydroxy-1,4-naphthoquinone (**1**) was selected as the model substrate to react with phenylacetylene (**2a**) at ambient temperature under irradiation of blue LEDs (460 nm). Product **3a** was obtained in 58% yield without any catalyst after 6 h irradiation in DCM (Table 1, entry 2). Furthermore, the use of different solvents, including acetone, THF, and toluene, failed to give better results than DCM (Table 1, entries 4–12, respectively). However, the yield was improved to 75% when a solvent of MeCN was used with irradiation for 6 h. (Table 1, entry 14). Consequently, the optimized reaction conditions turned out to be using MeCN as solvent under blue LEDs (460 nm) irradiation at ambient temperature for 6 h.

**Scheme 4:**
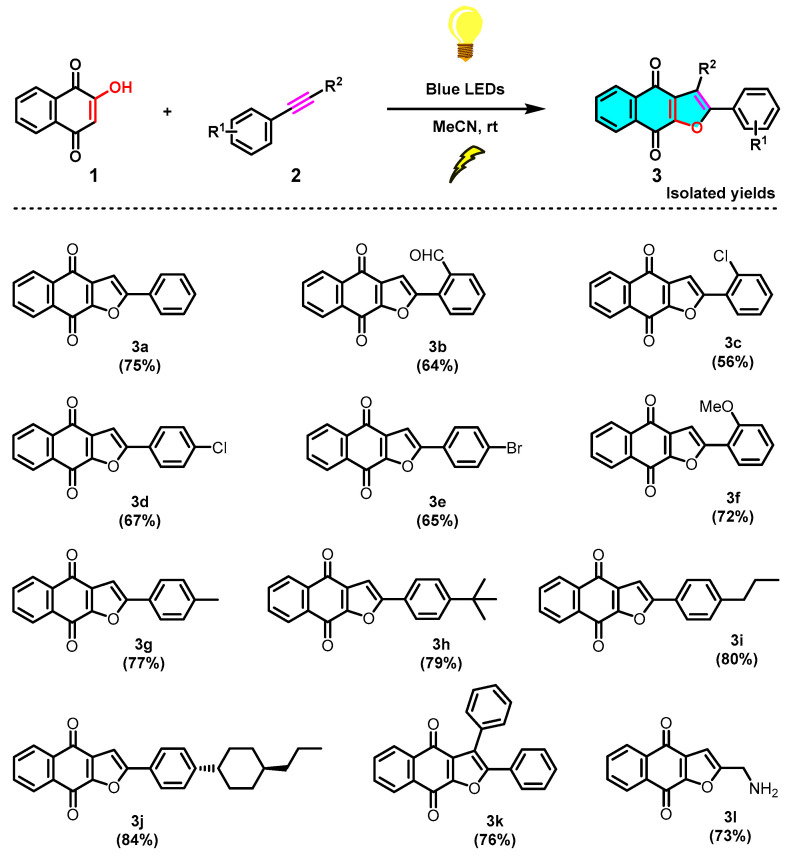
Scope of the photochemical synthesis of naphtho[2,3-*b*]furan-4,9-diones (**3**).

**Figure 1:**
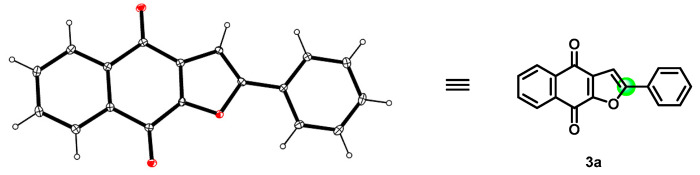
X-ray structure of compound **3a** (CCDC-2264554).

The structure of product **3a** was confirmed firstly by means of proton and carbon NMR spectra. It was noted that the naphtho[2,3-*b*]furan-4,9-dione 3a was obtained with complete regioselectivity, with only 2-phenylnaphtho[2,3-*b*]furan-4,9-dione was obtained and no isomer 3-phenylnaphtho[2,3-*b*]furan-4,9-dione being detectable or isolable (Scheme 3). Furthermore, the configuration of the main compound **3a** was unambiguously established by single-crystal X-ray diffraction analysis (Figure 1), which indicated that the phenyl group is at a C-2 position (green) instead of a C-3 position. With the optimized conditions in hand (Table 1, entry 14), a series of substituted phenylacetylenes **2a**−**2k** were evaluated (Scheme 4). It was noticed that the analogous reactions of formyl and various halogens substituted phenylacetylenes (**2b**–**2e**) with 2-hydroxy-1,4-naphthoquinone (**1**) successfully generated the corresponding products 3b-3e in moderate yields, respectively. Subsequently, we evaluated various alkyls substituted phenylacetylenes (**2g**–**2j**), such as Me, *t*Bu, and cyclohexyl, all of which favourably delivered the corresponding products **3g**–**3j** in good to very good yields, not showing significant differences in yield. Relatively speaking, an electron-donating group on the benzene ring, as in the case of **3j**, is more favorable than the electron-withdrawing groups (CHO and halogens) of **3b**–**3e**. Furthermore, other two phenylacetylene derivatives, diphenylacetylene and propargylamine (**2k** and **2l**), were evaluated too, and corresponding naphtho[2,3-*b*]furan-4,9-diones **3k** and **3l** were obtained swimmingly, as predicted, in good yields.

**Scheme 5:**
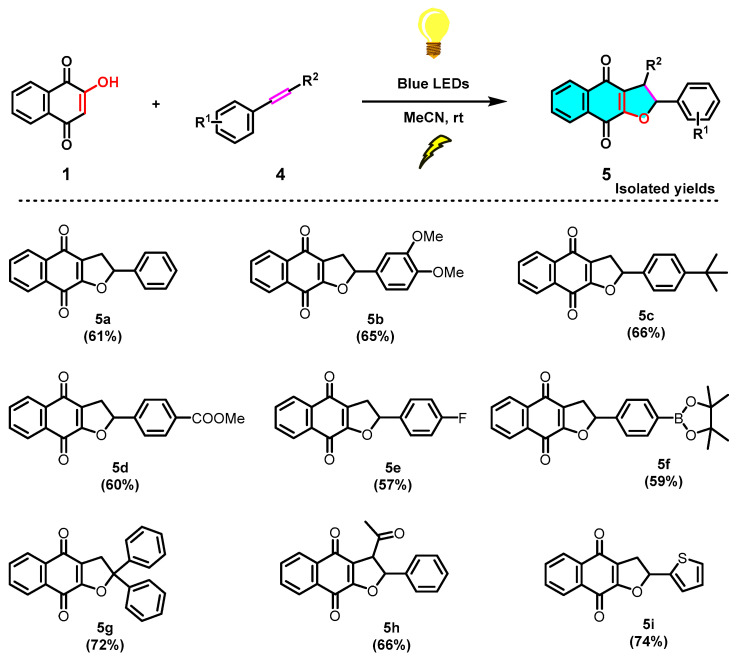
Scope of the photochemical synthesis of dihydronaphtho[2,3-*b*]furan-4,9-diones (**5**).

Next, to further examine the feasibility of this reaction, we examined the variation of the styrene component toward the formation of dihydronaphtho[2,3-*b*]furan-4,9-diones (**5**) (Scheme 5). With the above optimized conditions in hand (Table 1, entry 14), a series of substituted styrenes **4a**–**4h** were evaluated (Scheme 5). It was observed that the analogous reactions of EDG or EWG substituted styrenes with 2-hydroxy-1,4-naphthoquinone (**1**) smoothly produced the corresponding products **5a**–**5h** in moderate yields, respectively. Thereafter, we evaluated a heterocyclic olefin 2-vinylthiophene (**4i**), which was also found to be effective, as demonstrated in the successful installation of **5i** with a 74% yield. Overall, different substituted phenylacetylene derivatives (**2**) and styrene derivatives (**4**) reacted with 2-hydroxy-1,4-naphthoquinone (**1**) and generated corresponding cycloaddition products naphtho[2,3-*b*]furan-4,9-diones (**3**) and dihydronaphtho[2,3-*b*]furan-4,9-diones (**5**), not showing significant differences in yield.

**Scheme 6:**
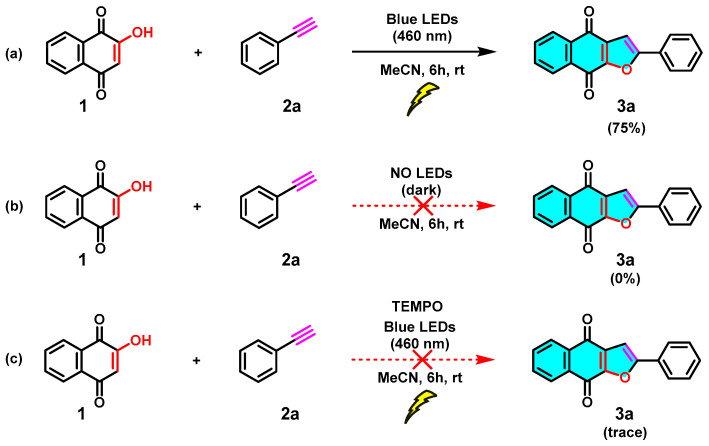
Control experiments for the photocatalyzed [3+2] cycloaddition reaction: (**a**) experiment was performed under standard conditions, (**b**) experiment was performed in the absence of light, (**c**) experiment was performed in the presence of TEMPO.

**Scheme 7:**
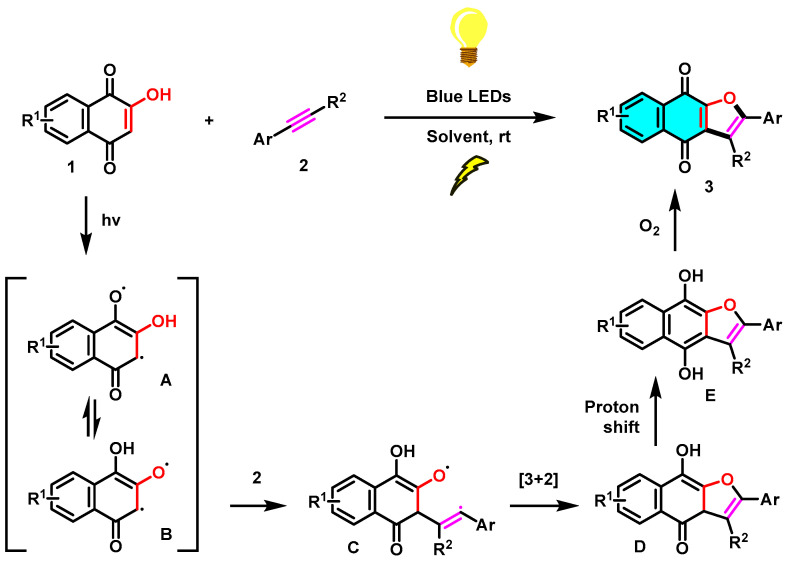
Proposed mechanism for the photocatalyzed [3+2] cycloaddition reaction.

To reveal the mechanism of this visible-light-mediated [3+2] cycloaddition reaction, several control experiments were performed (Scheme 6). As expected, no reaction occurs in the absence of light (Scheme 6b), which highlights the fact that the [3+2] cycloaddition reaction needs to be mediated by visible light. Furthermore, in reaction c of Scheme 6, TEMPO was added as a radical scavenger under standard conditions, and a trace amount of the target product **3a** was observed. Based on the control experimental results and the reported literature [28], a possible mechanism for this blue visible-light photocatalyzed [3+2] cycloaddition reaction was proposed, as shown in Scheme 7. First, the irradiation of **1** in MeCN generates tautomeric excited triplets (A) and (B), which react with an alkyne (**2**) to give a 1,5-biradical intermediate (C). Subsequently, an intramolecular [3+2] cyclization of the intermediate C gives hydroquinone intermediate (D). Upon 1,3-hydrogen transfer, the hydroquinone intermediate (E) is formed, and then, naphtho[2,3-*b*]furan-4,9-diones (**3**) is produced by air oxidation of the hydroquinone by oxygen in the air. Similarly, the [3+2] cycloaddition reaction of 2-hydroxy-1,4-naphthoquinone (**1**) with alkenes (**4**) leading to product dihydronaphtho[2,3-*b*]furan-4,9-diones (**5**) may proceed in a manner parallel to the [3+2] cycloaddition of alkynes and may also involve biradical intermediates.

## 3. Materials and Methods

### 3.1. General Information

All starting materials were purchased from available commercial suppliers and used without further purification. Thin layer chromatography (TLC) was performed on silica gel GF254 plates. Melting points were obtained by using an XT-5A digital melting-point apparatus and were uncorrected. The NMR spectra were recorded on a Bruker Avance 400 spectrometer at 400 MHz (^1^H NMR) and 100 MHz (^13^C NMR). HRMS analyses were carried out on a Thermo Fisher Q-Exactive mass spectrometer, which was operated in electrospray ionization (ESI) mode.

### 3.2. General Procedure for the Synthesis of Naphtho[2,3-b]furan-4,9-diones (***3***)

In a 25 mL tube, 2-hydroxy-1,4-naphthoquinone 1 (1.0 mmol) and alkyne 2 (1.0 mmol) were dissolved in 20 mL of acetonitrile. The reaction mixture was under irradiation of visible blue LEDs (460 nm) for 6.0 h. After completion (by TLC), the reaction mixture was evaporated to dryness in a vacuo. The residue was purified by medium-pressure chromatography (silica gel) using a mixed solvent of hexane and ethyl acetate (10–50% EA). The products were characterized by ^1^H NMR, ^13^C NMR, and HRMS spectroscopy.

*2-Phenylnaphtho[2,3-b]furan-4,9-dione (***3a***).* Yellow solid, yield 75%, m.p. 244–247 °C; ^1^H NMR (400 MHz, CDCl_3_): *δ* (ppm) 7.12 (s, 1H, Ar-*H*), 7.37–7.43 (m, 3H, Ar-*H*), 7.67–7.70 (m, 2H, Ar-*H*), 7.81–7.84 (m, 2H, Ar-*H*), 8.11–8.18 (m, 2H, Ar-*H*); ^13^C NMR (100 MHz, CDCl_3_): *δ* (ppm) 102.9, 125.6, 126.9, 127.0, 128.3, 130.3, 132.4, 132.9, 133.1, 133.6, 134.0, 151.6, 160.4, 173.1, 180.8; HRMS (ESI), *m*/*z* calcd 275.0803 for C_18_H_11_O_3_ [M + H]^+^, found 275.0807.

*2-(4,9-Dioxo-4,9-dihydronaphtho[2,3-b]furan-2-yl)benzaldehyde (***3b***).* Faint yellow solid, yield 64%, m.p. 188–191 °C; ^1^H NMR (400 MHz, CDCl_3_): *δ* (ppm) 7.28 (s, 1H, Ar-*H*), 7.64–7.68 (m, 1H, Ar-*H*), 7.74–7.82 (m, 3H, Ar-*H*), 7.89 (d, 1H, *J* = 7.6 Hz, Ar-*H*), 8.10 (dd, 1H, *J* = 7.6 Hz, 0.8 Hz, Ar-*H*), 8.24–8.29 (m, 2H, Ar-*H*), 10.44 (s, 1H, C*H*O); ^13^C NMR (100 MHz, CDCl_3_): *δ* (ppm) 108.8, 127.1, 127.2, 129.4, 129.8, 130.4, 132.6, 132.6, 133.0, 133.9, 134.0, 134.1, 134.2, 152.8, 158.2, 173.3, 180.5, 190.6; HRMS (ESI), *m*/*z* calcd 303.0652 for C_19_H_11_O_4_ [M + H]^+^, found 303.0655.

*2-(2-Chlorophenyl)naphtho[2,3-b]furan-4,9-dione (***3c***).* Orange-yellow, yield 56%, m.p. 191–194 °C; ^1^H NMR (400 MHz, CDCl_3_): *δ* (ppm) 7.36–7.44 (m, 2H, Ar-*H*), 7.51–7.54 (m, 1H, Ar-*H*), 7.68 (s, 1H, Ar-*H*), 7.76–7.78 (m, 2H, Ar-*H*), 8.09 (dd, 1H, *J* = 8.0 Hz, 2.0 Hz, Ar-*H*), 8.21–8.26 (m, 2H, Ar-*H*); ^13^C NMR (100 MHz, CDCl_3_): *δ* (ppm) 108.5, 126.9, 127.1, 127.3, 129.3, 130.7, 131.9, 132.0, 132.8, 133.2, 133.8, 134.0, 151.3, 156.4, 173.2, 180.7; HRMS (ESI), *m*/*z* calcd 309.0313 for C_18_H_10_ClO_3_ [M + H]^+^, found 309.0317.

*2-(4-Chlorophenyl)naphtho[2,3-b]furan-4,9-dione (***3d***).* Orange solid, yield 67%, m.p. 209–212 °C; ^1^H NMR (400 MHz, CDCl_3_): *δ* (ppm) 7.19 (s, 1H, Ar-*H*), 7.62 (d, 2H, *J* = 8.4 Hz, Ar-*H*), 7.74–7.78 (m, 4H, Ar-*H*), 8.18–8.25 (m, 2H, Ar-*H*); ^13^C NMR (100 MHz, CDCl_3_): *δ* (ppm) 103.5, 124.7, 126.9, 126.9, 127.0, 127.2, 132.3, 132.4, 132.8, 133.1, 133.7, 134.1, 151.7, 159.2, 173.0, 180.6; HRMS (ESI), *m*/*z* calcd 309.0313 for C_18_H_10_ClO_3_ [M + H]^+^, found 309.0316.

*2-(4-Bromophenyl)naphtho[2,3-b]furan-4,9-dione (***3e***).* Yellow solid, yield 65%, m.p. 264–266 °C; ^1^H NMR (400 MHz, CDCl_3_): *δ* (ppm) 7.12 (s, 1H, Ar-*H*), 7.37–7.43 (m, 3H, Ar-*H*), 7.67–7.70 (m, 2H, Ar-*H*), 7.81–7.84 (m, 2H, Ar-*H*), 8.11–8.18 (m, 2H, Ar-*H*); ^13^C NMR (100 MHz, CDCl_3_): *δ* (ppm) 55.6, 107.7, 111.2, 117.3, 121.0, 126.8, 126.9, 127.5, 131.2, 132.6, 133.0, 133.2, 133.5, 133.9, 150.5, 157.0, 173.0, 181.2; HRMS (ESI), *m*/*z* calcd 352.9808 for C_18_H_10_BrO_3_ [M + H]^+^, found 352.9810.

*2-(2-Methoxyphenyl)naphtho[2,3-b]furan-4,9-dione (***3f***).* Yellow solid, yield 72%, m.p. 207–209 °C; ^1^H NMR (400 MHz, CDCl_3_): *δ* (ppm) 4.03 (s, 3H, OC*H*_3_), 7.05 (d, 1H, *J* = 8.4 Hz, Ar-*H*), 7.12 (t, 1H, *J* = 7.2 Hz, Ar-*H*), 7.41–7.45 (m, 1H, Ar-*H*), 7.52 (s, 1H, Ar-*H*), 7.75–7.78 (m, 2H, Ar-*H*), 8.13–8.27 (m, 3H, Ar-*H*); ^13^C NMR (100 MHz, CDCl_3_): *δ* (ppm) 103.4, 124.7, 126.9, 127.0, 127.2, 132.3, 132.4, 132.8, 133.1, 133.6, 134.1, 151.7, 159.2, 173.1, 180.6; HRMS (ESI), *m*/*z* calcd 305.0808 for C_19_H_13_O_4_ [M + H]^+^, found 305.0812.

*2-(p-Tolyl)naphtho[2,3-b]furan-4,9-dione (***3g***).* Faint yellow solid, yield 77%, m.p. 260–263 °C; ^1^H NMR (400 MHz, CDCl_3_): *δ* (ppm) 2.39 (s, 3H, C*H*_3_), 7.11 (s, 1H, Ar-*H*), 7.27 (d, 2H, *J* = 7.6 Hz, Ar-*H*), 7.72–7.77 (m, 4H, Ar-*H*), 8.16–8.21 (m, 2H, Ar-*H*); ^13^C NMR (100 MHz, CDCl_3_): *δ* (ppm) 21.5, 102.3, 125.5, 126.8, 126.9, 129.8, 132.5, 132.9, 133.1, 133.5, 133.9, 134.7, 160.6, 172.9, 180.9; HRMS (ESI), *m*/*z* calcd 289.0859 for C_19_H_13_O_3_ [M + H]^+^, found 289.0861.

*2-(4-(Tert-butyl)phenyl)naphtho[2,3-b]furan-4,9-dione (***3h***).* Faint yellow solid, yield 79%, m.p. 180–182 °C; ^1^H NMR (400 MHz, CDCl_3_): *δ* (ppm) 1.36 (s, 9H, C(C*H*_3_)_3_), 7.15 (s, 1H, Ar-*H*), 7.50–7.52 (m, 2H, Ar-*H*), 7.73–7.84 (m, 4H, Ar-*H*), 8.18–8.25 (m, 2H, Ar-*H*); ^13^C NMR (100 MHz, CDCl_3_): *δ* (ppm) 31.2, 35.0, 102.4, 125.4, 125.6, 126.1, 126.9, 126.9, 132.6, 133.0, 133.1, 133.5, 133.9, 151.4, 153.9, 160.9, 173.0, 180.9; HRMS (ESI), *m*/*z* calcd 331.1329 for C_22_H_19_O_3_ [M + H]^+^, found 331.1333.

*2-(4-Propylphenyl)naphtho[2,3-b]furan-4,9-dione (***3i***).* Yellow solid, yield 80%, m.p. 166–169 °C; ^1^H NMR (400 MHz, CDCl_3_): *δ* (ppm) 0.99 (t, 3H, *J* = 7.6 Hz, C*H*_3_), 1.61–1.73 (m, 2H, C*H*_2_), 2.66 (t, 2H, *J* = 7.6 Hz, C*H*_2_), 7.16 (s, 1H, Ar-*H*), 7.31 (d, 2H, *J* = 8.0 Hz, Ar-*H*), 7.75–7.84 (m, 4H, Ar-*H*), 8.20–8.27 (m, 2H, Ar-*H*); ^13^C NMR (100 MHz, CDCl_3_): *δ* (ppm) 13.8, 24.3, 38.0, 102.3, 125.6, 126.8, 126.9, 126.9, 129.2, 132.6, 132.9, 145.5, 160.8, 172.9, 180.9; HRMS (ESI), *m*/*z* calcd 317.1172 for C_21_H_17_O_3_ [M + H]^+^, found 317.1175.

*2-(4-((1s,4r)-4-Propylcyclohexyl)phenyl)naphtho[2,3-b]furan-4,9-dione (***3j***).* Yellow solid, yield 84%, m.p. 179–182 °C; ^1^H NMR (400 MHz, CDCl_3_): *δ* (ppm) 0.91 (t, 3H, *J* = 7.6 Hz, C*H*_3_), 1.05–1.08 (m, 2H, C*H*_2_), 1.22–1.25 (m, 2H, C*H*_2_), 1.32–1.37 (m, 3H, C*H*_2_, C*H*), 1.46–1.50 (m, 2H, C*H*_2_), 1.88–1.93 (m, 4H, C*H*_2_), 2.50–2.56 (m, 1H, C*H*), 7.14 (s, 1H, Ar-*H*), 7.33 (d, 2H, *J* = 8.0 Hz, Ar-*H*), 7.74–7.76 (m, 2H, Ar-*H*), 7.81 (d, 2H, *J* = 8.0 Hz, Ar-*H*), 8.18–8.25 (m, 2H, Ar-*H*); ^13^C NMR (100 MHz, CDCl_3_): *δ* (ppm) 14.4, 20.0, 33.4, 33.4, 37.0, 39.7, 44.6, 102.3, 125.6, 125.9, 126.9, 126.9, 127.6, 132.6, 133.0, 133.1, 133.5, 133.9, 150.7, 151.3, 160.8, 172.9, 180.9; HRMS (ESI), *m*/*z* calcd 399.1955 for C_27_H_27_O_3_ [M + H]^+^, found 399.1957.

*2,3-Diphenylnaphtho[2,3-b]furan-4,9-dione (***3k***).* Orange solid, yield 76%, m.p. 262–265 °C; ^1^H NMR (400 MHz, CDCl_3_): *δ* (ppm) 7.37–7.43 (m, 3H, Ar-*H*), 7.35–7.43 (m, 8H, Ar-*H*), 7.59–7.63 (m, 1H, Ar-*H*), 7.77 (d, 1H, *J* = 7.6 Hz, Ar-*H*), 8.00 (dd, 1H, *J* = 8.0 Hz, 0.4 Hz, Ar-*H*); ^13^C NMR (100 MHz, CDCl_3_): *δ* (ppm) 121.8, 121.8, 122.4, 126.6, 128.4, 128.5, 128.6, 128.7, 128.9, 129.0, 129.1, 129.9, 130.2, 130.2, 130.4, 135.4, 151.5, 159.2, 174.5, 180.6; HRMS (ESI), *m*/*z* calcd 351.1016 for C_24_H_15_O_3_ [M + H]^+^, found 351.1019.

*2-(Aminomethyl)naphtho[2,3-b]furan-4,9-dione (***3l***).* Faint yellow solid, yield 73%, m.p. 217–219 °C; ^1^H NMR (400 MHz, CDCl_3_): *δ* (ppm) 3.71 (d, 2H, *J* = 2.4 Hz, C*H*_2_), 5.47 (s, 1H, Ar-*H*), 7.56 (t, 1H, *J* = 7.2 Hz, Ar-*H*), 7.67 (t, 1H, *J* = 7.6 Hz, Ar-*H*), 7.80 (d, 1H, *J* = 7.6 Hz, Ar-*H*), 7.85 (d, 1H, *J* = 7.6 Hz, Ar-*H*),; ^13^C NMR (100 MHz, CDCl_3_): *δ* (ppm) 29.1, 77.6, 107.6, 125.3, 125.5, 130.9, 132.1, 133.8, 135.8, 181.3; HRMS (ESI), *m*/*z* calcd 228.0655 for C_13_H_10_NO_3_ [M + H]^+^, found 228.0655.

### 3.3. General Procedure for the Synthesis of Dihydronaphtho[2,3-b]furan-4,9-diones (***5***)

In a 25 mL tube, 2-hydroxy-1,4-naphthoquinone 1 (1.0 mmol) and alkenes 4 (1.0 mmol) were dissolved in 20 mL of acetonitrile. The reaction mixture was under irradiation of visible blue LEDs (460 nm) for 6.0 h. After completion (by TLC), the reaction mixture was evaporated to dryness in vacuo. The residue was purified by medium-pressure chromatography (silica gel) using a mixed solvent of hexane and ethyl acetate (10–50% EA). The products were characterized by ^1^H NMR, ^13^C NMR, and HRMS spectroscopy.

*2-Phenyl-2,3-dihydronaphtho[2,3-b]furan-4,9-dione (***5a***).* Yellow solid, yield 61%, m.p. 171–173 °C; ^1^H NMR (400 MHz, CDCl_3_): *δ* (ppm) 3.27 (dd, 1H, *J* = 16.8 Hz, 8.4 Hz, C*H*_2_); 3.67 (dd, 1H, *J* = 17.2 Hz, 10.8 Hz, C*H*_2_); 6.00 (dd, 1H, *J* = 10.8 Hz, 8.8 Hz, C*H*); 7.39–7.41 (m, 4H, Ar-*H*), 7.46 (s, 1H, Ar-*H*), 7.69–7.74 (m, 2H, Ar-*H*), 8.08–8.12 (m, 2H, Ar-*H*); ^13^C NMR (100 MHz, CDCl_3_): *δ* (ppm) 35.3, 86.8, 123.9, 126.0, 126.1, 126.4, 128.9, 131.6, 133.1, 133.1, 134.2, 139.5, 159.9, 177.7, 182.2; HRMS (ESI), *m*/*z* calcd 277.0859 for C_18_H_13_O_3_ [M + H]^+^, found 277.0861.

*2-(3,4-Dimethoxyphenyl)-2,3-dihydronaphtho[2,3-b]furan-4,9-dione (***5b***).* Faint yellow solid, yield 65%, m.p. 177–179 °C; ^1^H NMR (400 MHz, CDCl_3_): *δ* (ppm) 3.28 (dd, 1H, *J* = 17.2 Hz, 9.2 Hz, C*H*_2_); 3.62 (dd, 1H, *J* = 17.2 Hz, 10.8 Hz, C*H*_2_); 3.88 (s, 6H, OC*H*_3_); 5.94 (dd, 1H, *J* = 8.4 Hz, 7.2 Hz, C*H*); 6.87 (d, 1H, *J* = 8.0 Hz, Ar-*H*); 6.92–6.98 (m, 2H, Ar-*H*), 7.68–7.73 (m, 2H, Ar-*H*), 8.07–8.09 (m, 2H, Ar-*H*); ^13^C NMR (100 MHz, CDCl_3_): *δ* (ppm) 34.8, 56.0, 56.0, 87.1, 109.5, 111.2, 119.0, 123.9, 126.0, 126.3, 131.6, 133.0, 133.1, 134.2, 149.4, 149.7, 159.7, 177.7, 182.2; HRMS (ESI), *m*/*z* calcd 336.0992 for C_20_H_16_O_5_ [M + H]^+^, found 336.0995.

*2-(4-(Tert-butyl)phenyl)-2,3-dihydronaphtho[2,3-b]furan-4,9-dione (***5c***).* Yellow solid, yield 66%, m.p. 166–169 °C; ^1^H NMR (400 MHz, CDCl_3_): *δ* (ppm) 1.32 (s, 9H, C(C*H*_3_)_3_), 3.30 (dd, 1H, *J* = 17.2 Hz, 8.8 Hz, C*H*_2_); 3.64 (dd, 1H, *J* = 17.2 Hz, 10.8 Hz, C*H*_2_); 5.98 (dd, 1H, *J* = 10.4 Hz, 8.4 Hz, C*H*), 7.34 (d, 2H, *J* = 8.4 Hz, Ar-*H*), 7.42 (d, 2H, *J* = 8.4 Hz, Ar-*H*), 7.69–7.75 (m, 2H, Ar-*H*), 8.01–8.12 (m, 2H, Ar-*H*),; ^13^C NMR (100 MHz, CDCl_3_): *δ* (ppm) 29.7, 31.3, 34.9, 86.8, 123.9, 124.0, 125.8, 125.9, 126.1, 126.4, 127.8, 131.7, 133.0, 134.2, 136.4, 152.2, 159.9, 182.2; HRMS (ESI), *m*/*z* calcd 333.1485 for C_22_H_21_O_3_ [M + H]^+^, found 333.1488.

*Methyl 4-(4,9-dioxo-2,3,4,9-tetrahydronaphtho[2,3-b]furan-2-yl)benzoate (***5d***).* Yellow solid, yield 60%, m.p. 168–171 °C; ^1^H NMR (400 MHz, CDCl_3_): *δ* (ppm) 3.22 (dd, 1H, *J* = 17.2 Hz, 8.8 Hz, C*H*_2_); 3.72 (dd, 1H, *J* = 17.2 Hz, 10.8 Hz, C*H*_2_); 3.92 (s, 3H, OC*H*_3_), 6.05 (dd, 1H, *J* = 10.8 Hz, 8.4 Hz, C*H*); 7.48 (d, 2H, *J* = 8.4 Hz, Ar-*H*), 7.68–7.76 (m, 2H, Ar-*H*), 8.06–8.11 (m, 4H, Ar-*H*); ^13^C NMR (100 MHz, CDCl_3_): *δ* (ppm) 35.5, 52.2, 85.8, 123.7, 125.7, 126.1, 126.4, 130.2, 131.6, 133.0, 133.2, 134.3, 144.5, 159.7, 166.5, 177.5, 182.0; HRMS (ESI), *m*/*z* calcd 334.0841 for C_20_H_14_O_5_ [M + H]^+^, found 334.0844.

*2-(4-Fluorophenyl)-2,3-dihydronaphtho[2,3-b]furan-4,9-dione (***5e***).* Faint yellow solid, yield 57%, m.p. 175–177 °C; ^1^H NMR (400 MHz, CDCl_3_): *δ* (ppm) 3.23 (dd, 1H, *J* = 17.2 Hz, 8.4 Hz, C*H*_2_); 3.66 (dd, 1H, *J* = 17.2 Hz, 10.8 Hz, C*H*_2_); 5.98 (dd, 1H, *J* = 10.4 Hz, 8.8 Hz, C*H*); 7.06–7.09 (m, 2H, Ar-*H*), 7.37–7.41 (m, 2H, Ar-*H*), 7.70–7.76 (m, 2H, Ar-*H*), 8.08–8.11 (m, 2H, Ar-*H*); ^13^C NMR (100 MHz, CDCl_3_): *δ* (ppm) 35.3, 86.1, 115.8, 116.0, 123.7, 126.1, 126.4, 127.9, 131.6, 133.0, 133.1, 134.3, 159.7, 177.7, 182.1; HRMS (ESI), *m*/*z* calcd 295.0765 for C_18_H_12_FO_3_ [M + H]^+^, found 295.0769.

*2-(4-(4,4,5,5-Tetramethyl-1,3,2-dioxaborolan-2-yl)phenyl)-2,3-dihydronaphtho[2,3-b]furan-4,9-dione (***5f***).* Yellow solid, yield 59%, m.p. 170–172 °C; ^1^H NMR (400 MHz, CDCl_3_): *δ* (ppm) 1.35 (s, 12H, C*H*_3_), 3.23 (dd, 1H, *J* = 17.2 Hz, 8.8 Hz, C*H*_2_); 3.67 (dd, 1H, *J* = 17.2 Hz, 10.8 Hz, C*H*_2_); 6.01 (dd, 1H, *J* = 10.8 Hz, 8.8 Hz, C*H*); 7.48 (d, 2H, *J* = 8.4 Hz, Ar-*H*), 7.69–7.75 (m, 2H, Ar-*H*), 7.84 (d, 2H, *J* = 8.4 Hz, Ar-*H*), 8.08–8.12 (m, 2H, Ar-*H*); ^13^C NMR (100 MHz, CDCl_3_): *δ* (ppm) 24.9, 35.4, 84.0, 86.6, 123.9, 125.0, 126.1, 126.4, 131.6, 133.1, 133.1, 134.2, 135.4, 142.5, 159.9, 177.7, 182.2; HRMS (ESI), *m*/*z* calcd 403.1711 for C_24_H_24_BO_5_ [M + H]^+^, found 403.1713.

*2,2-Diphenyl-2,3-dihydronaphtho[2,3-b]furan-4,9-dione (***5g***).* Faint yellow solid, yield 72%, m.p. 171–173 °C; ^1^H NMR (400 MHz, CDCl_3_): *δ* (ppm) 3.92 (s, 2H, C*H*_2_), 7.28–7.36 (m, 6H, Ar-*H*), 7.45–7.48 (m, 4H, Ar-*H*), 7.64–7.68 (m, 2H, Ar-*H*), 8.02–8.09 (m, 2H, Ar-*H*); ^13^C NMR (100 MHz, CDCl_3_): *δ* (ppm) 41.6, 96.4, 123.7, 125.8, 126.0, 126.4, 128.2, 128.6, 131.7, 133.0, 134.1, 143.6, 158.6, 177.6, 182.2; HRMS (ESI), *m*/*z* calcd 353.1172 for C_24_H_17_O_3_ [M + H]^+^, found 353.1175.

*3-Acetyl-2-phenyl-2,3-dihydronaphtho[2,3-b]furan-4,9-dione (***5h***).* Yellow solid, yield 66%, m.p. 195–197 °C; ^1^H NMR (400 MHz, CDCl_3_): *δ* (ppm) 2.54 (s, 3H, C*H*_3_), 4.52 (d, 1H, *J* = 7.2 Hz, C*H*); 6.27 (d, 1H, *J* = 6.8 Hz, C*H*); 7.31–7.36 (m, 2H, Ar-*H*), 7.37–7.40 (m, 3H, Ar-*H*), 7.72–7.76 (m, 2H, Ar-*H*), 8.07–8.13 (m, 2H, Ar-*H*); ^13^C NMR (100 MHz, CDCl_3_): *δ* (ppm) 31.0, 60.9, 88.8, 122.0, 126.0, 125.6, 126.3, 126.6, 129.1, 129.3, 131.5, 132.9, 133.4, 134.5, 138.5, 160.4, 177.5, 181.7; HRMS (ESI), *m*/*z* calcd 319.0965 for C_20_H_15_O_4_ [M + H]^+^, found 319.0968.

*2-(Thiophen-2-yl)-2,3-dihydronaphtho[2,3-b]furan-4,9-dione (***5i***).* Orange solid, yield 74%, m.p. 176–179 °C; ^1^H NMR (400 MHz, CDCl_3_): *δ* (ppm) 3.42 (dd, 1H, *J* = 17.6 Hz, 8.8 Hz, C*H*_2_); 3.67 (dd, 1H, *J* = 17.2 Hz, 10.8 Hz, C*H*_2_); 6.00 (dd, 1H, *J* = 10.0 Hz, 8.4 Hz, C*H*); 7.01–7.04 (m, 1H, Ar-*H*), 7.19–7.20 (m, 1H, Ar-*H*), 7.36–7.38 (m, 1H, Ar-*H*), 7.68–7.75 (m, 2H, Ar-*H*), 8.07–8.10 (m, 2H, Ar-*H*); ^13^C NMR (100 MHz, CDCl_3_): *δ* (ppm) 35.3, 82.5, 123.6, 126.1, 126.4, 127.0, 127.1, 131.6, 133.0, 133.1, 134.2, 141.7, 159.1, 177.6, 182.1; HRMS (ESI), *m*/*z* calcd 283.0423 for C_16_H_11_O_3_S [M + H]^+^, found 283.0425.

The results of the X-ray diffraction analysis for compounds **3a** and **5d** were deposited with the Cambridge Crystallographic Data Centre (CCDC 2264554 and 2264555).

## 4. Conclusions

In conclusion, visible-light-mediated [3+2] cycloaddition reactions of 2-hydroxy-1,4-naphthoquinones and alkynes and alkenes under irradiation of blue LEDs (460 nm) in the absence of any bases, metals, ligands, or other catalysts have been demonstrated. Under environmentally friendly conditions, a variety of naphtho[2,3-*b*]furan-4,9-diones and dihydronaphtho[2,3-*b*]furan-4,9-diones were delivered within 6 h in comparable or sometimes even (slightly) better yields than those presented in the literature. This green and efficient protocol shows excellent regioselectivity and remarkable functional group tolerance. This work provides a powerful, green, efficient, and facile means to expand the structural diversity of naphtho[2,3-*b*]furan-4,9-diones and dihydronaphtho[2,3-*b*]furan-4,9-diones as promising scaffolds for novel drug discovery.

## Figures and Tables

**Table 1:**
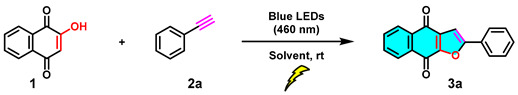
Optimization of the reaction conditions *^a^*.

Entry	Solvent	Time	Yield *^b^*
1	DCM	3 h	34%
2	DCM	6 h	58%
3	DCM	9 h	49%
4	Acetone	3 h	37%
5	Acetone	6 h	49%
6	Acetone	9 h	46%
7	THF	3 h	44%
8	THF	6 h	56%
9	THF	9 h	51%
10	Toluene	3 h	26%
11	Toluene	6 h	38%
12	Toluene	9 h	33%
13	MeCN	3 h	62%
14	MeCN	6 h	75%
15	MeCN	9 h	71%

*^a^* The reactions were carried out on a 1 mmol scale in 20 mL of solvent at room temperature. *^b^* Isolated yields.

## Data Availability

The data presented in this study are contained in the article tables and Supplementary Materials.

## Supplementary Materials

The following supporting information can be downloaded at: https://www.mdpi.com/article/10.3390/molecules28124751/s1, including ^1^H, ^13^C NMR, and ORTEP spectra of [3+2] cycloaddition products naphtho[2,3-*b*]furan-4,9-diones (**3**) and dihydronaphtho[2,3-*b*]furan-4,9-diones (**5**).

## Sample Availability

Samples of compounds **3** and **5** are available from the authors.
