# Supplementary material for: An Efficient Synthesis of Naphtho[2,3-b]furan-4,9-diones via Visible-Light-Mediated [3+2] Cycloaddition Reaction
Source: Molecules. 2023 Jun 13;28(12):4751. doi: 10.3390/molecules28124751 (PMC10301255; doi:10.3390/molecules28124751)
Supplement: Supplementary file 1 [file molecules-28-04751-s001.zip › molecules-2449171-SI.pdf]

Supporting Information

An Efficient Synthesis of Naphtho[2,3-*b*]furan-4,9-diones via Visible-Light-Mediated [3+2] Cycloaddition Reaction

Hongbo Tan [1,2,3,*], Zehui Qi [1], Yuanhui Yu [1], Xu Zhang [1], Yuheng Xiang [1], Jingwen Huang [1] and Zhigang Xu [1], Dianyong Tang [1], Zhongzhu Chen [1], Bochu Wang [2]

1 National & Local Joint Engineering Research Center of Targeted and Innovative Therapeutics, Chongqing Engineering Laboratory of Targeted and Innovative Therapeutics, Chongqing Key Laboratory of Kinase Modulators as Innovative Medicine, Chongqing Collaborative Innovation Center of Targeted and Innovative Therapeutics, College of Pharmacy & IATTI, Chongqing University of Arts and Sciences, Chongqing 402160, China

2 Key Laboratory of Biorheological Science and Technology, Ministry of Education, College of Bioengineering, Chongqing University, Chongqing 400030, China

3 Chongqing Academy of Chinese Materia Medica, Chongqing 400065, China

* Correspondence: 13167859296@163.com (Hongbo Tan)

Content **Page #**

^{1}H NMR and ^{13}C NMR spectrum of compound **3a-3l**. 1

^{1}H NMR and ^{13}C NMR spectrum of compound **5a-5i**. 13

ORTEP diagrams of crystal structures of **3a** and **5d**. 22

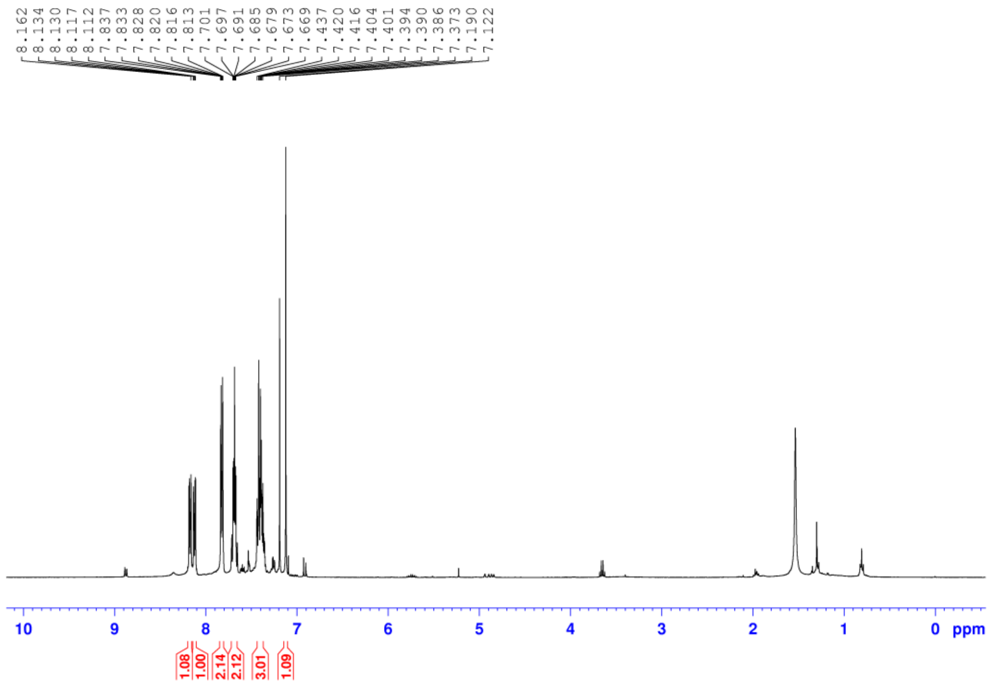

SI-Figure S1. ^{1}H NMR spectrum of compound **3a**.

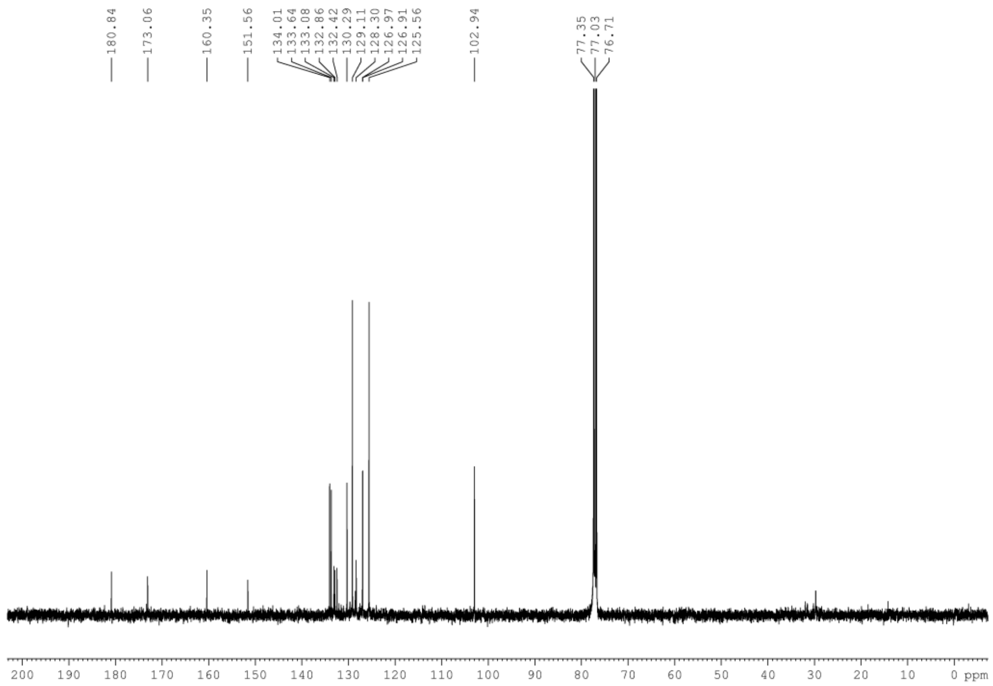

SI-Figure S2. ^{13}C NMR spectrum of compound **3a**.

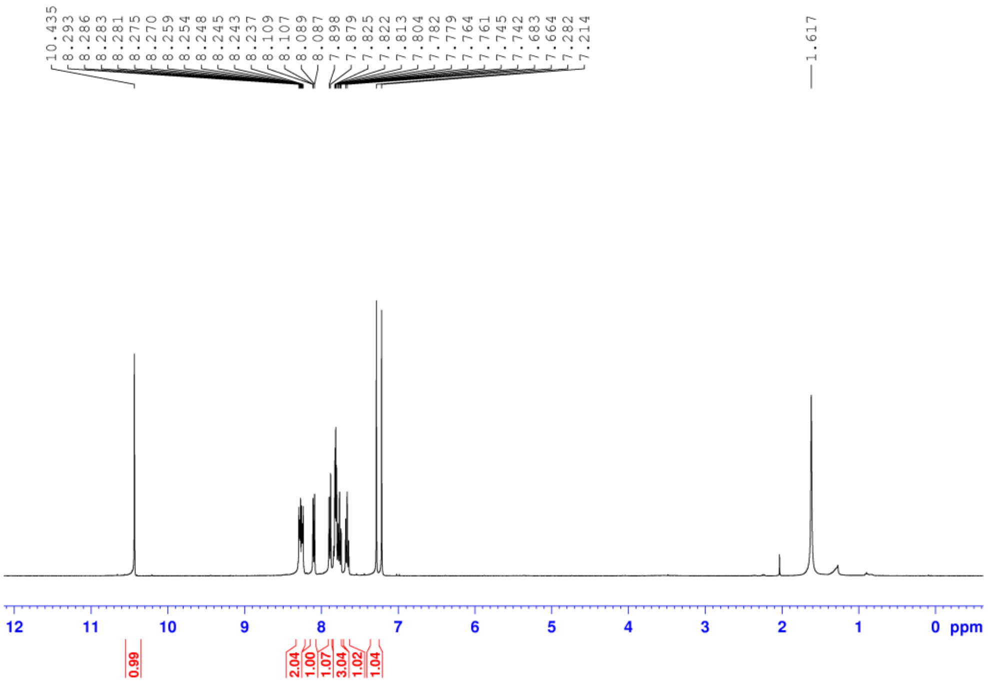

SI-Figure S3. ^{1}H NMR spectrum of compound **3b**.

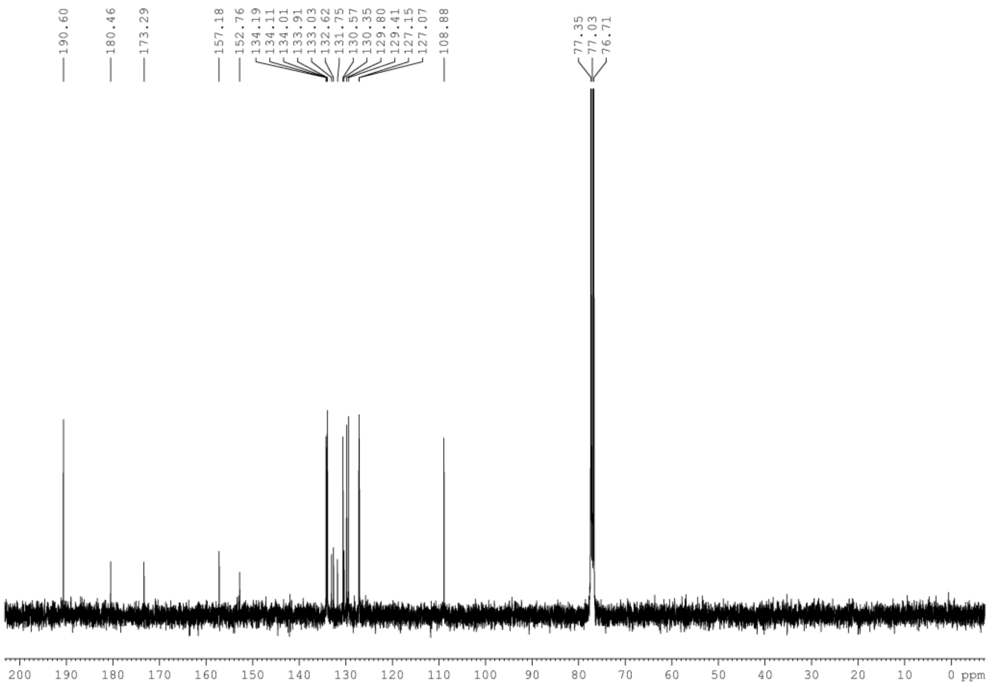

SI-Figure S4. ^{13}C NMR spectrum of compound **3b**.

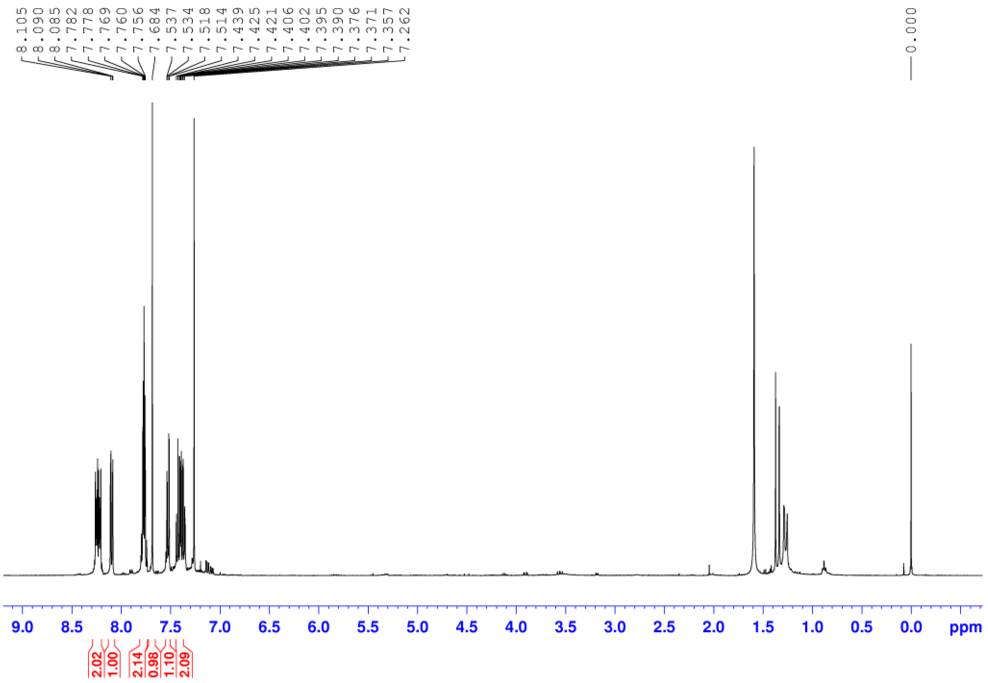

SI-Figure S5. ^{1}H NMR spectrum of compound **3c**.

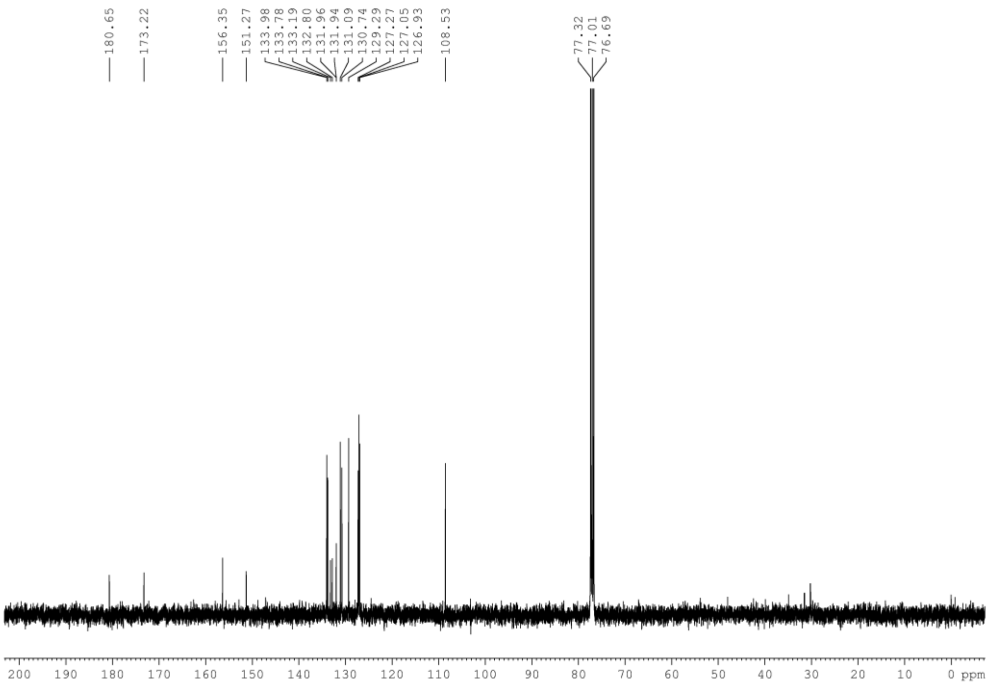

SI-Figure S6. ^{13}C NMR spectrum of compound **3c**.

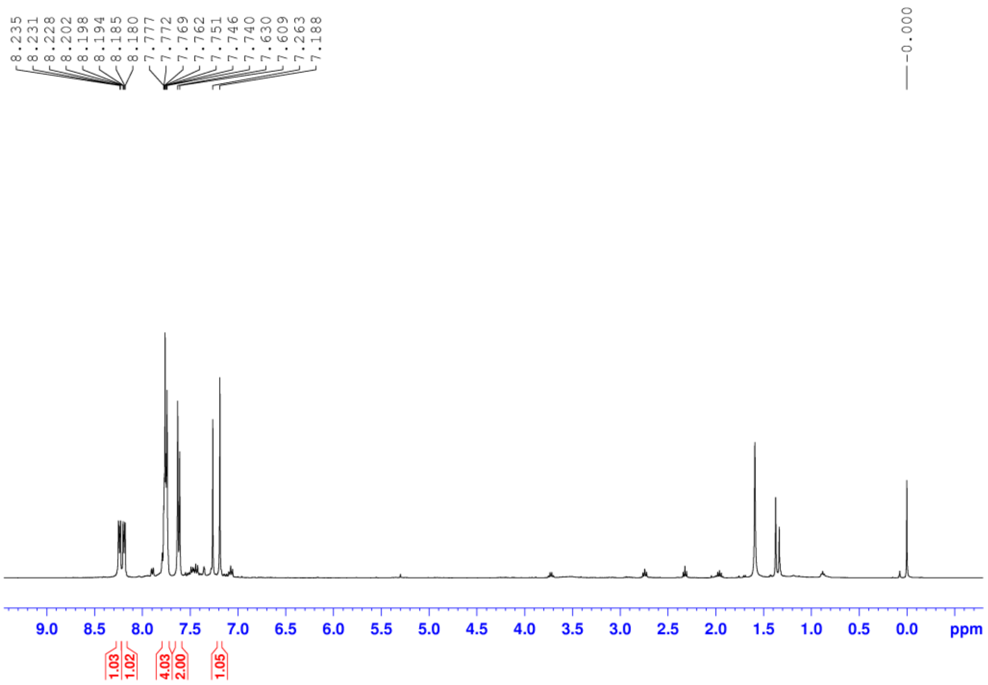

SI-Figure S7. ^{1}H NMR spectrum of compound **3d**.

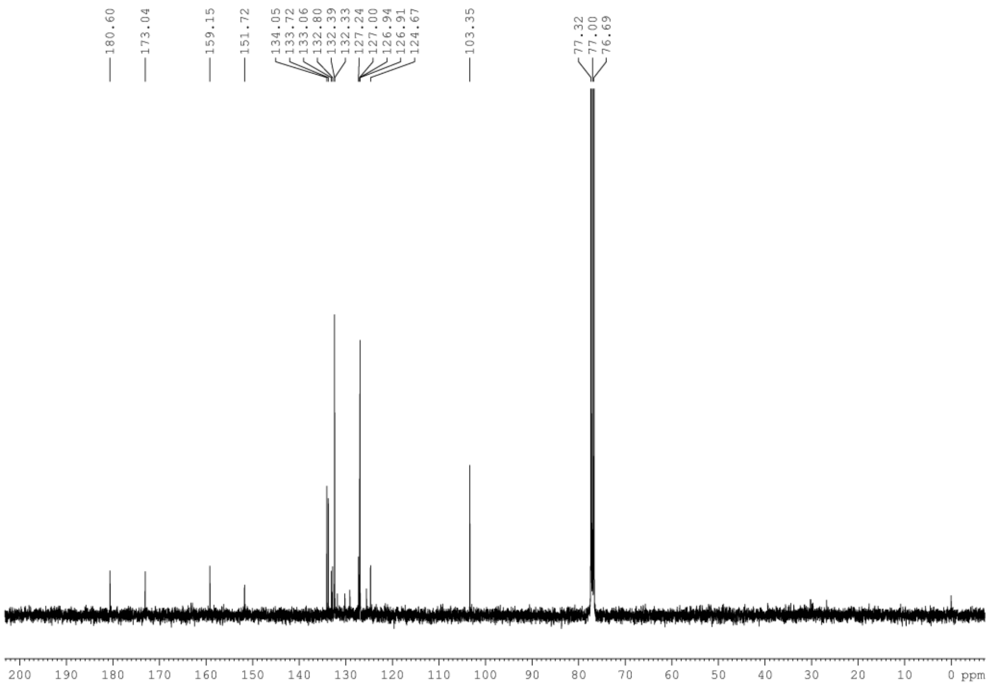

SI-Figure S8. ^{13}C NMR spectrum of compound **3d**.

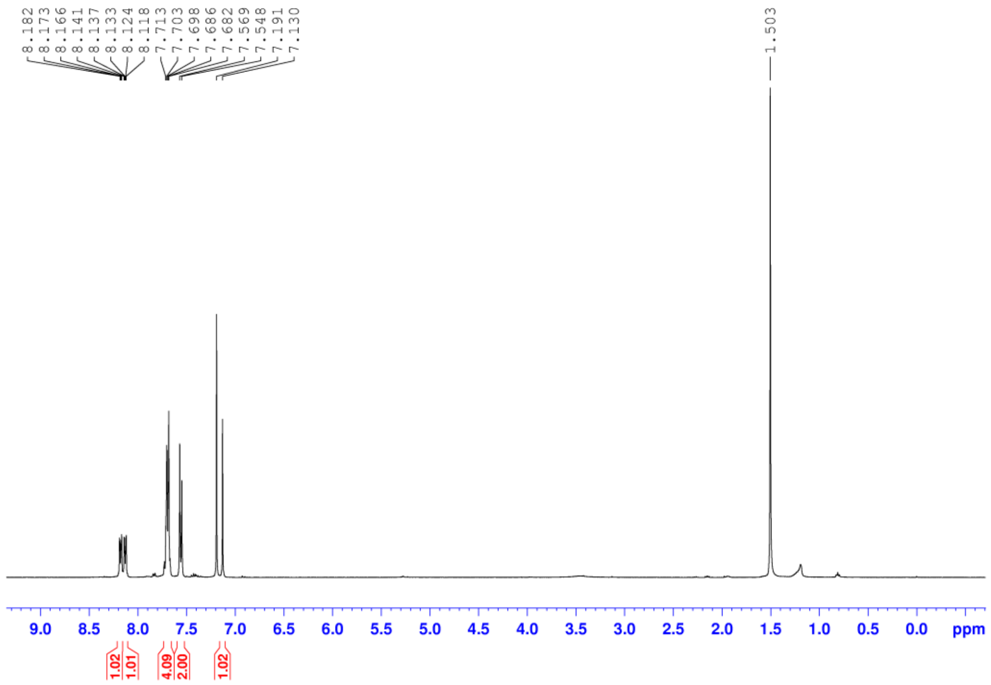

SI-Figure S9. ^{1}H NMR spectrum of compound **3e**.

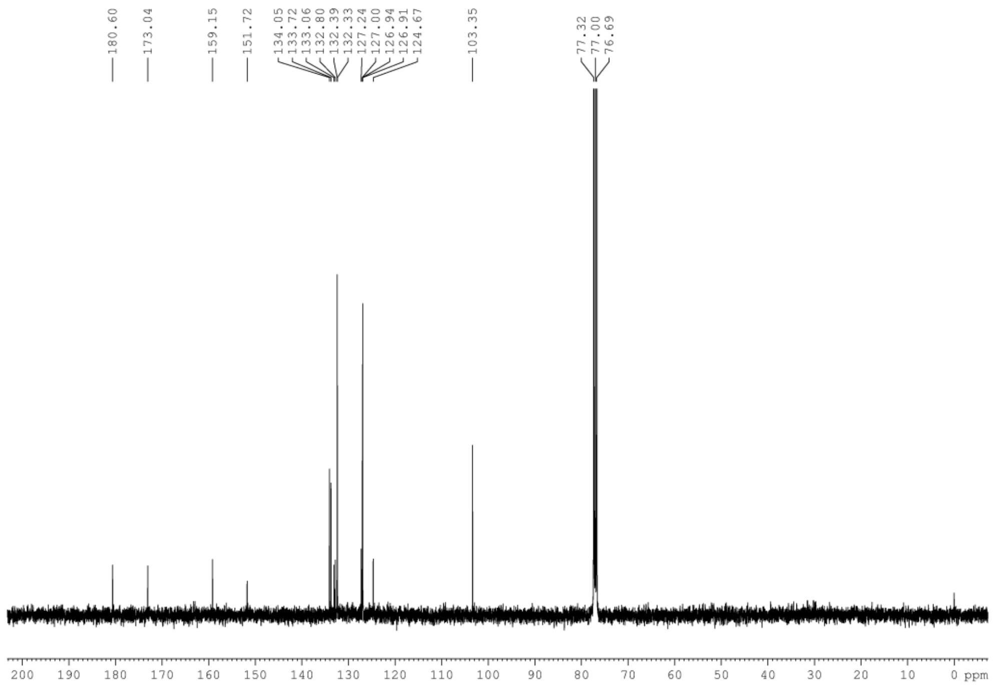

SI-Figure S10. ^{13}C NMR spectrum of compound **3e**.

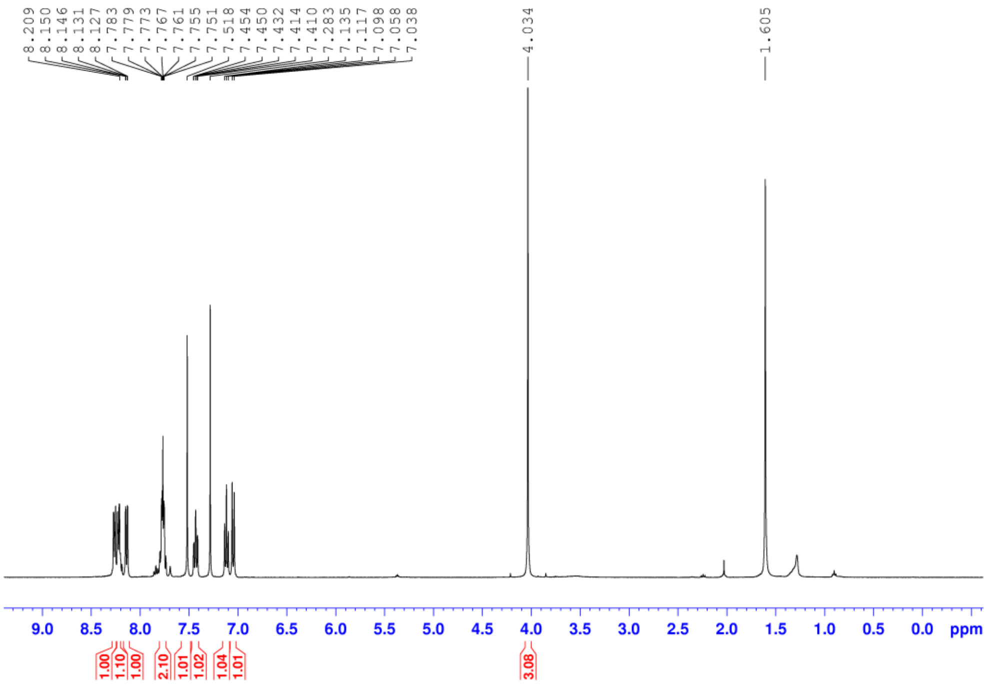

SI-Figure S11. ^{1}H NMR spectrum of compound **3f**.

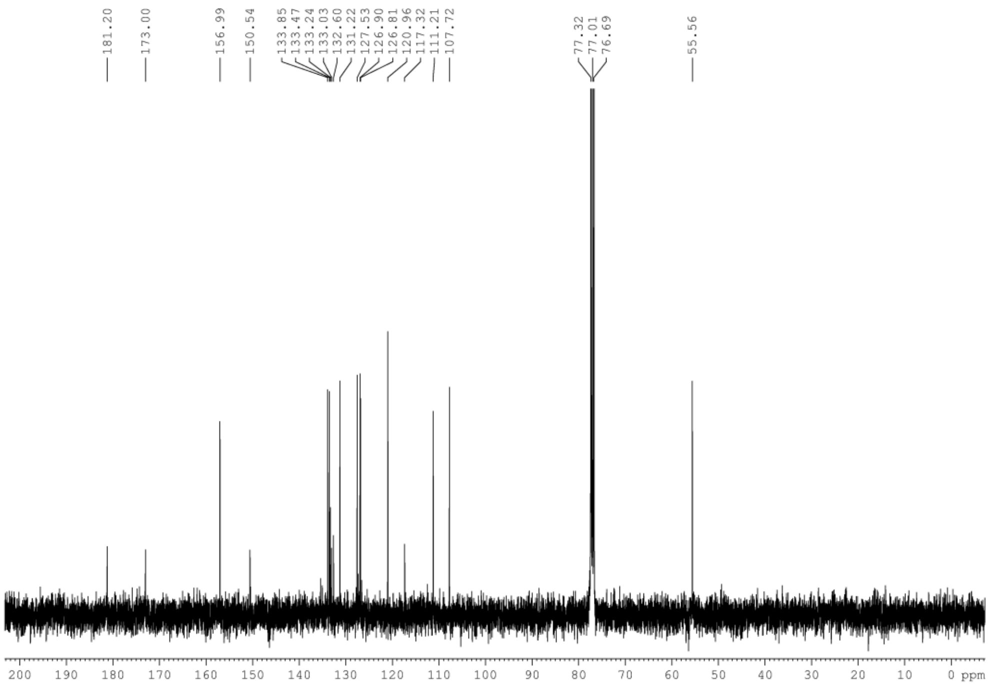

SI-Figure S12. ^{13}C NMR spectrum of compound **3f**.

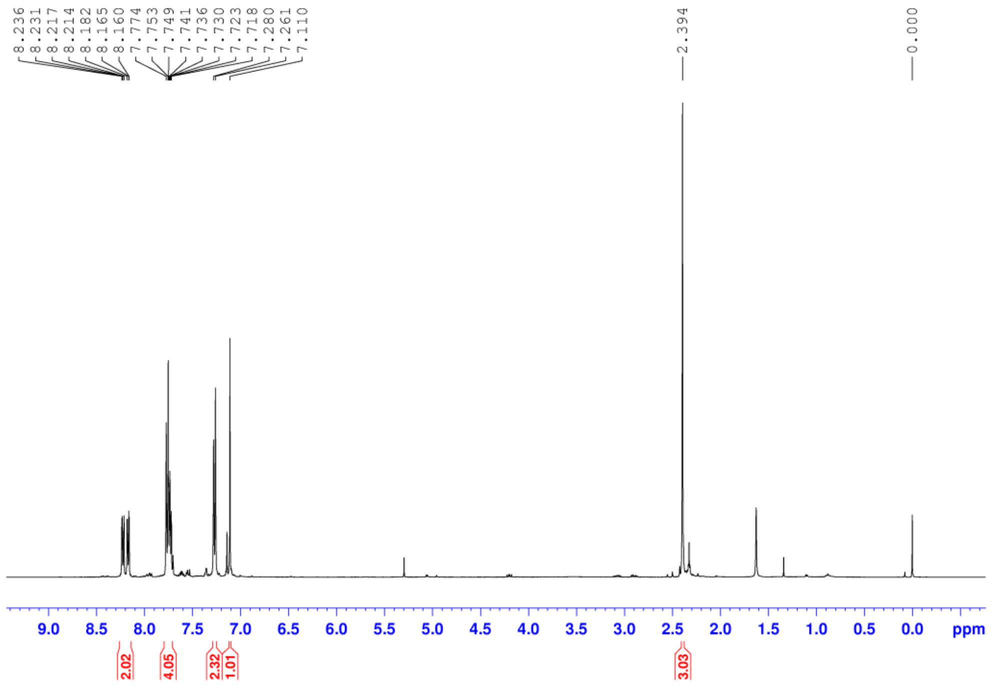

SI-Figure S13. ^{1}H NMR spectrum of compound **3g**.

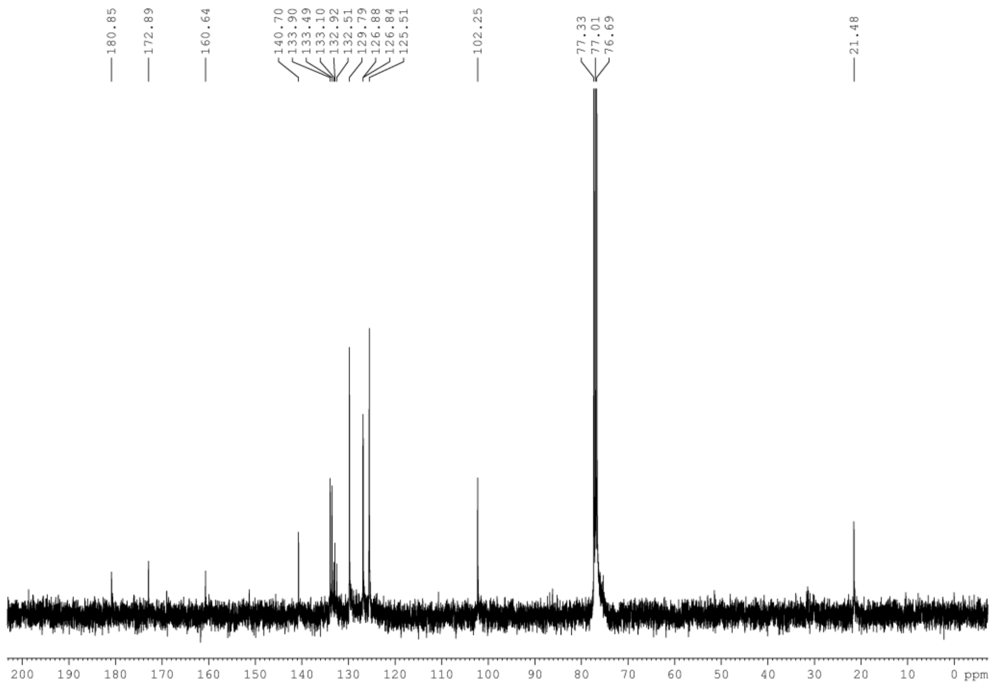

SI-Figure S14. ^{13}C NMR spectrum of compound **3g.**

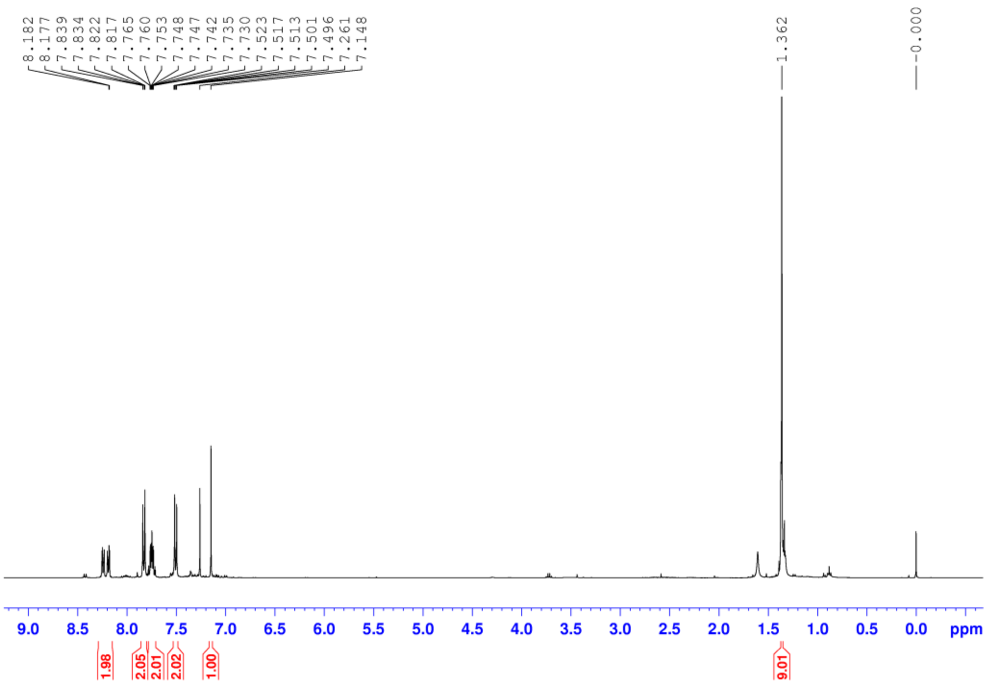

SI-Figure S15. ^{1}H NMR spectrum of compound **3h**.

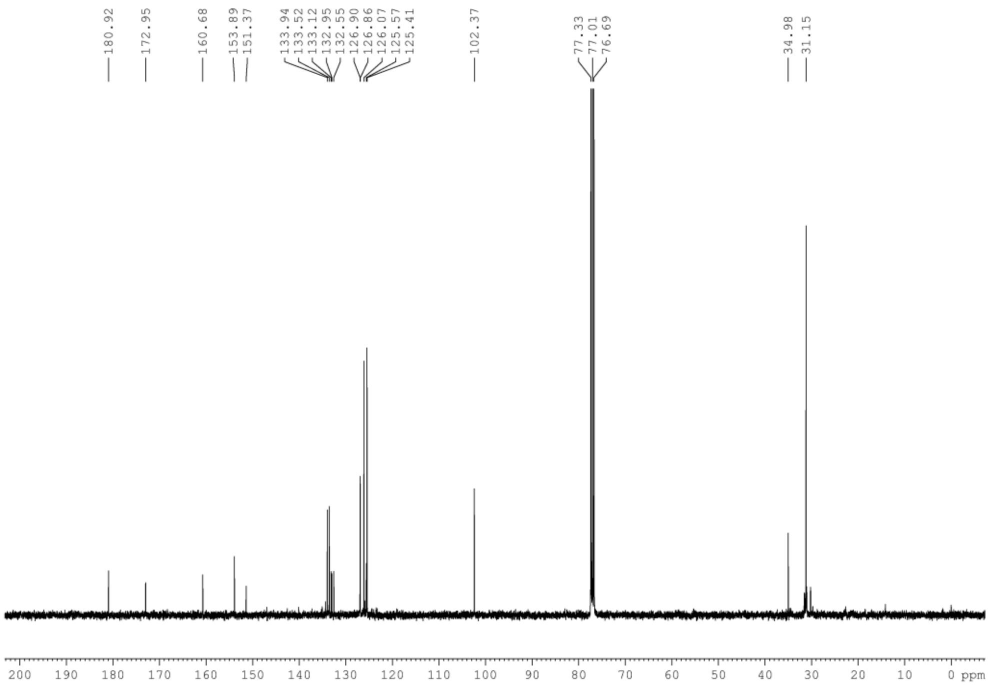

SI-Figure S16. ^{13}C NMR spectrum of compound **3h**.

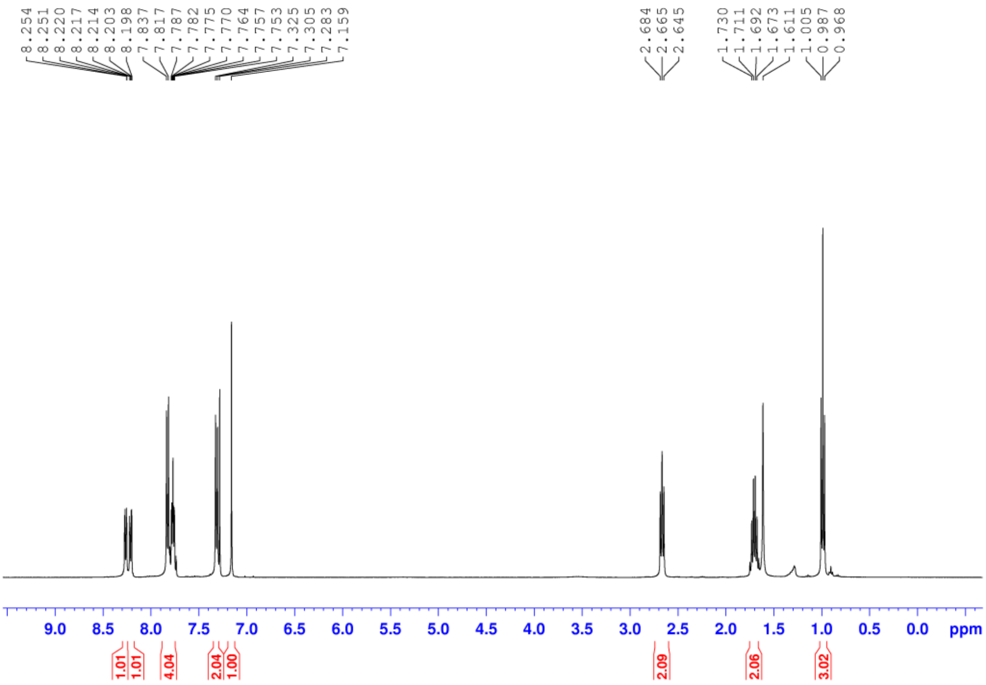

SI-Figure S17. ^{1}H NMR spectrum of compound **3i**.

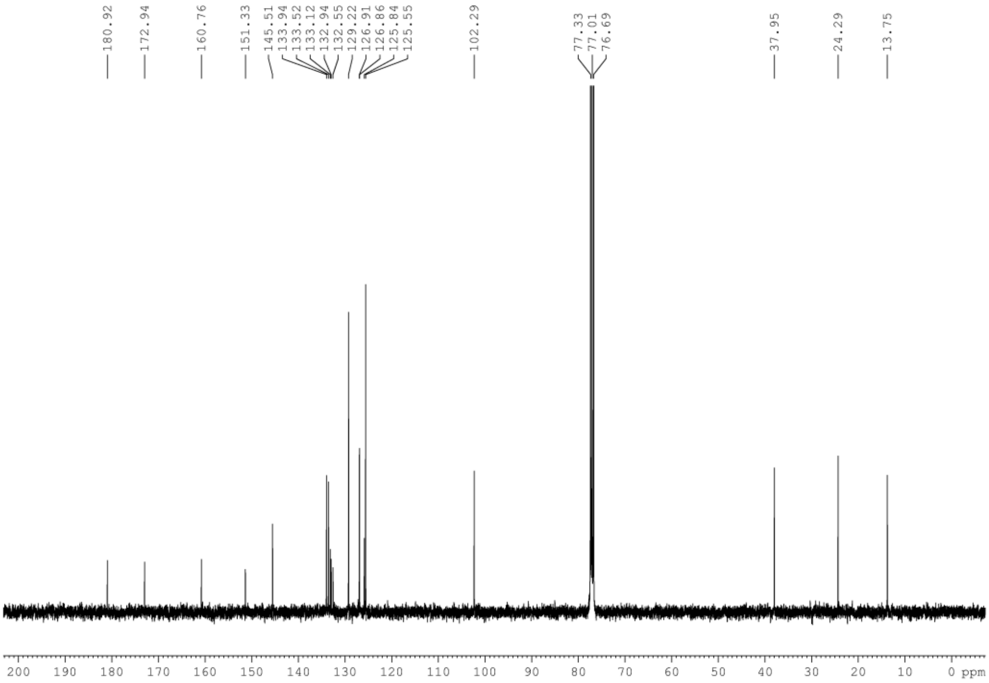

SI-Figure S18. ^{13}C NMR spectrum of compound **3i**.

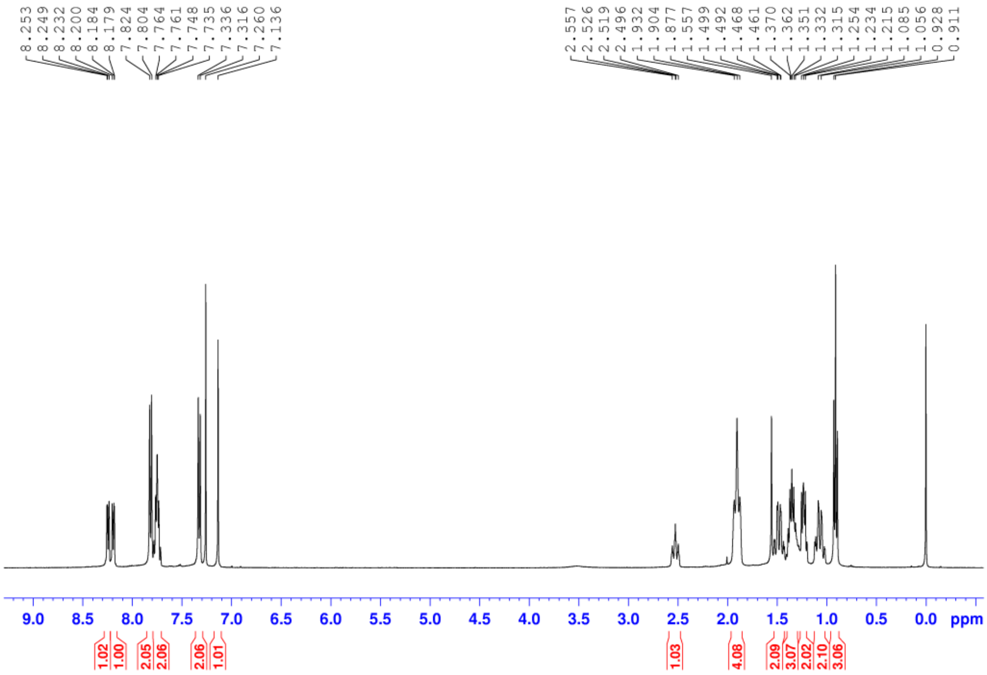

SI-Figure S19. ^{1}H NMR spectrum of compound **3j**.

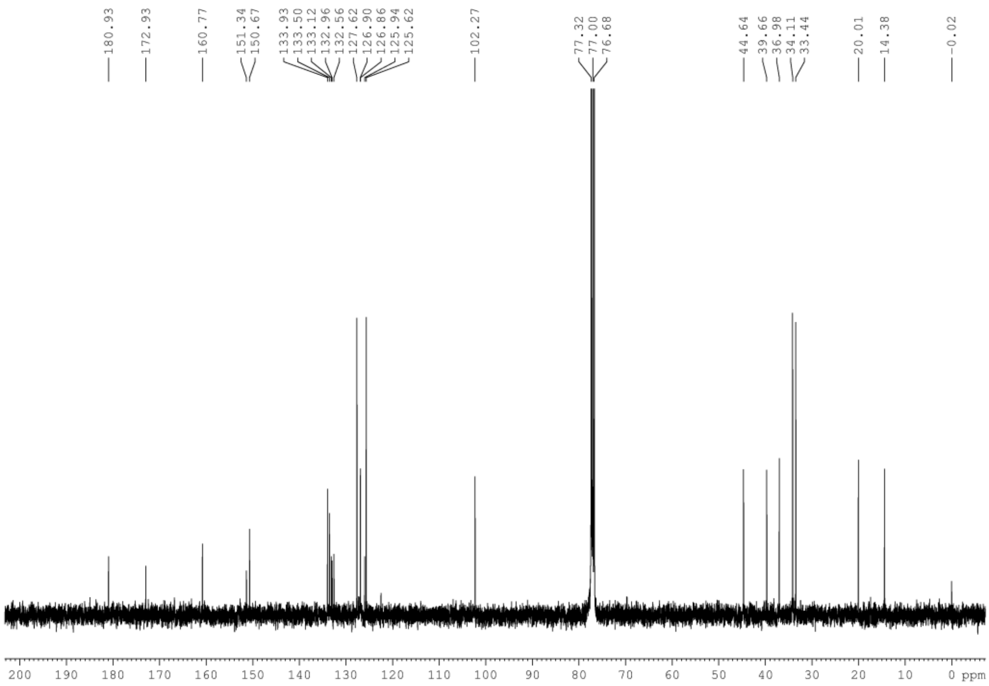

SI-Figure S20. ^{13}C NMR spectrum of compound **3j**.

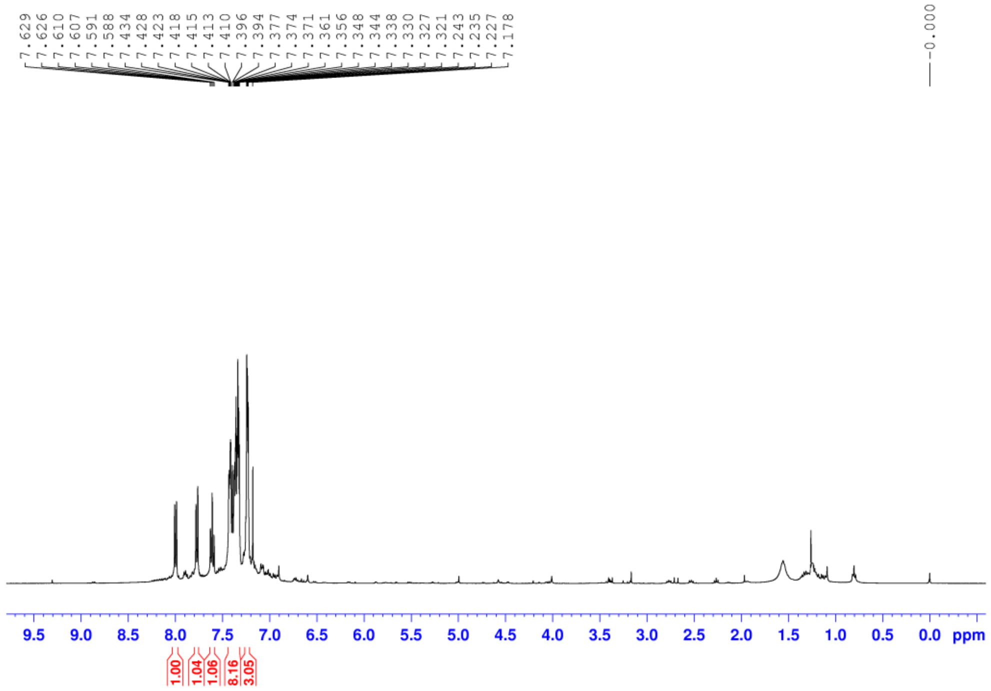

SI-Figure S21. ^{1}H NMR spectrum of compound **3k**.

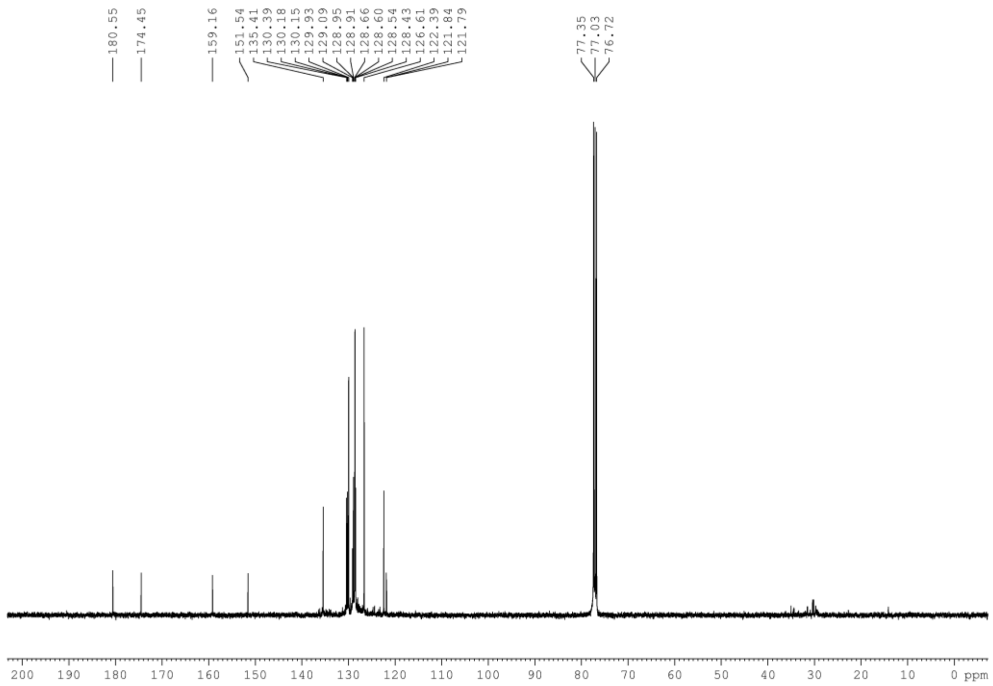

SI-Figure S22. ^{13}C NMR spectrum of compound **3k**.

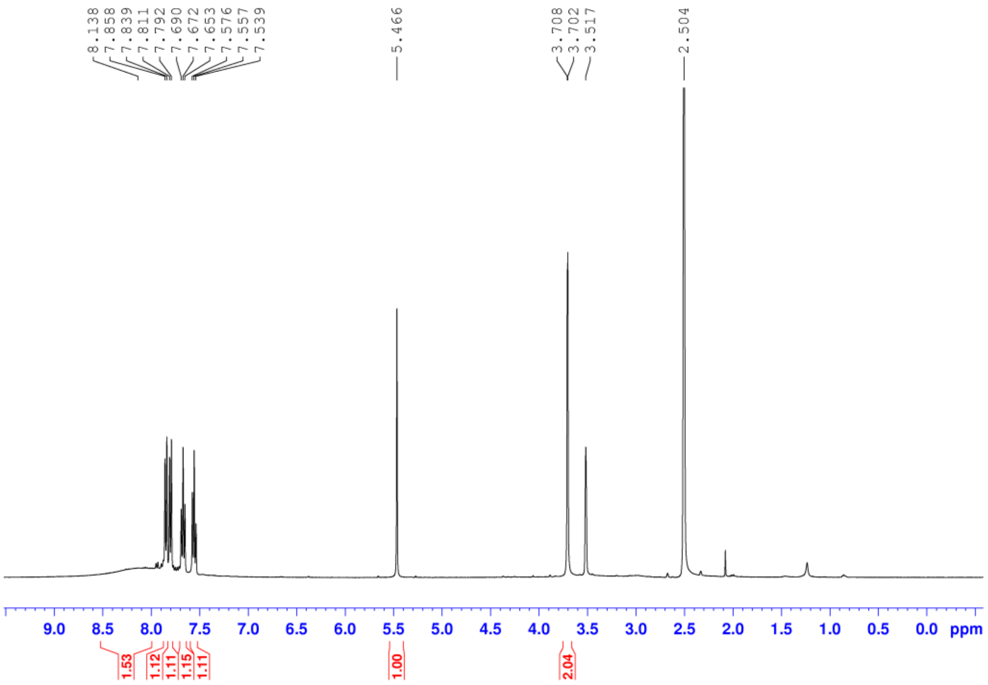

SI-Figure S23. ^{1}H NMR spectrum of compound **3l**.

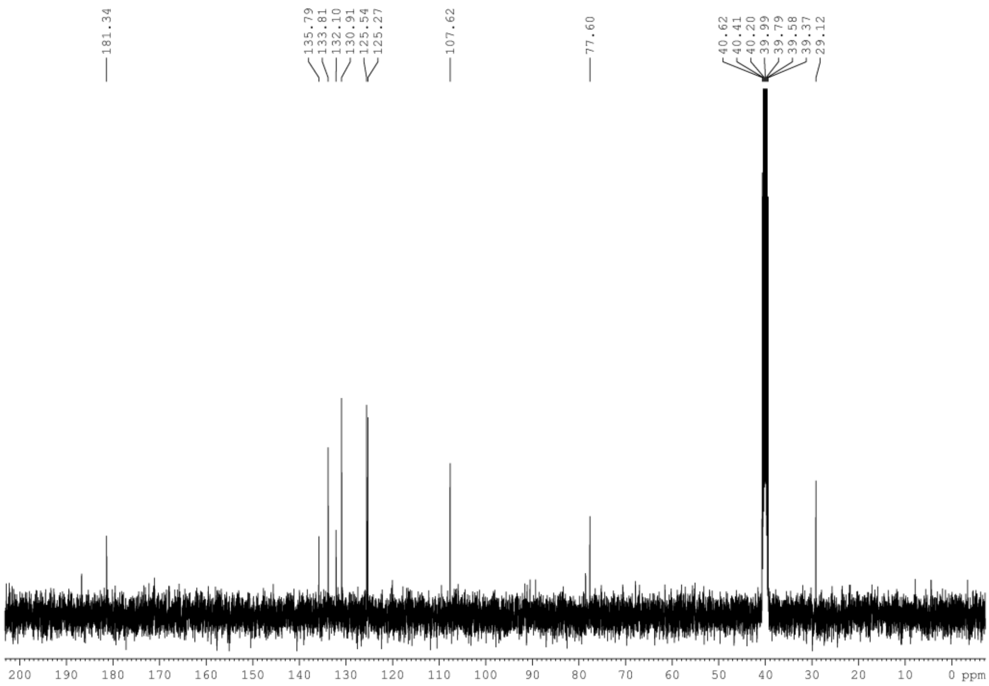

SI-Figure S24. ^{13}C NMR spectrum of compound **3l**.

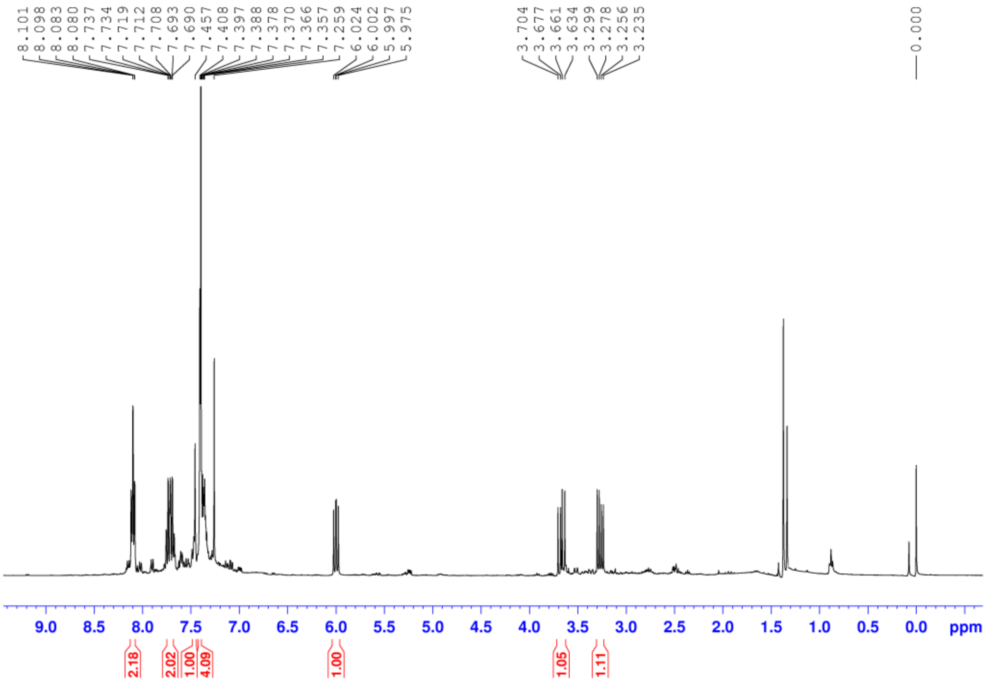

SI-Figure S25. ^{1}H NMR spectrum of compound **5a**.

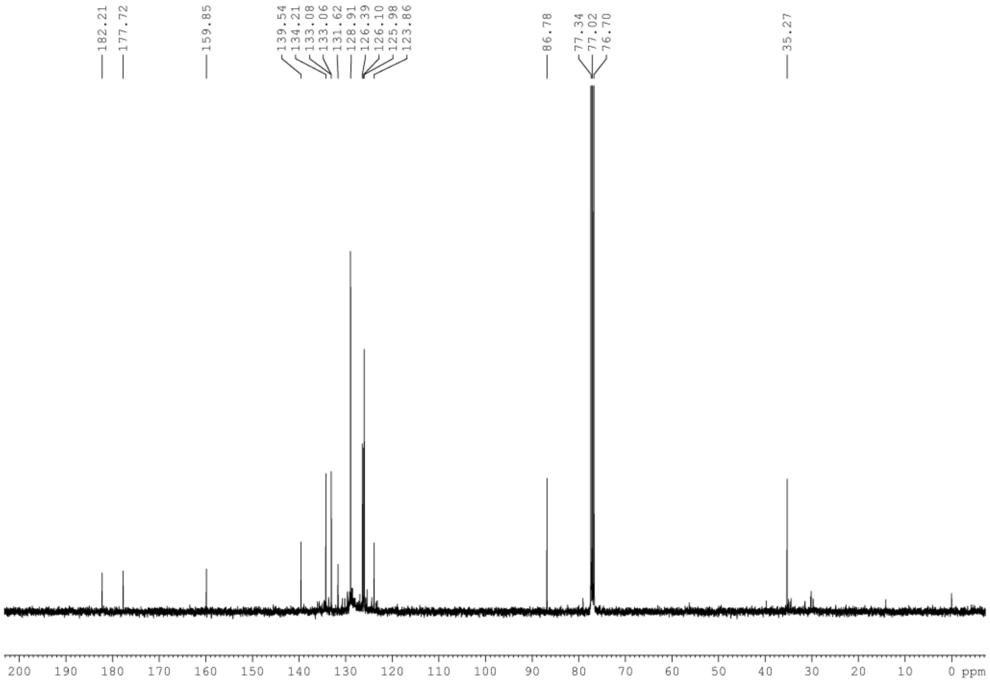

SI-Figure S26. ^{13}C NMR spectrum of compound **5a**.

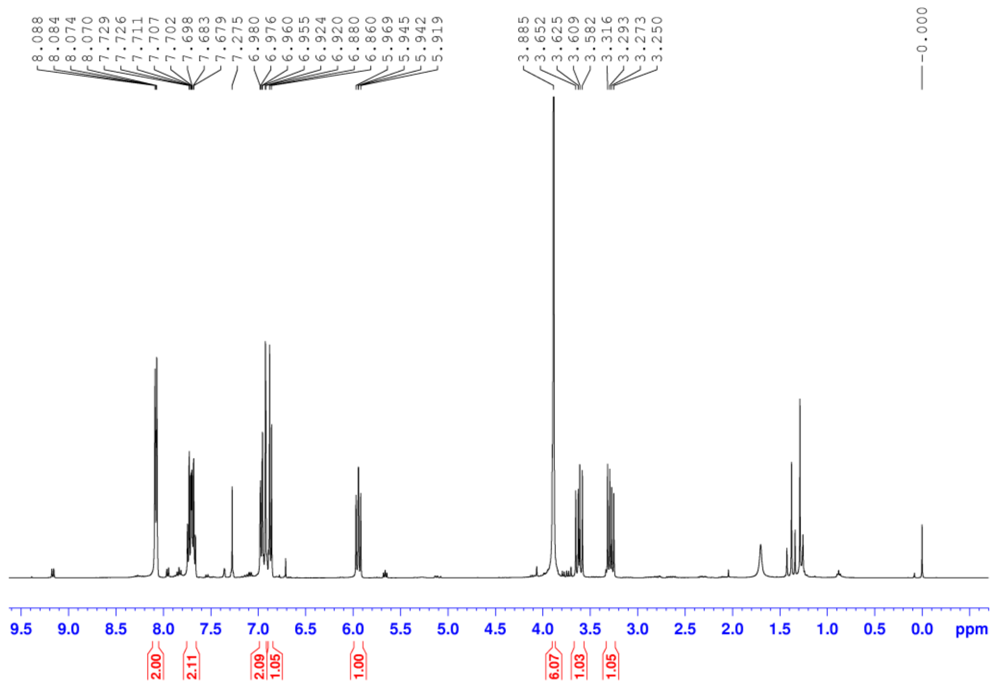

SI-Figure S27. ^{1}H NMR spectrum of compound **5b**.

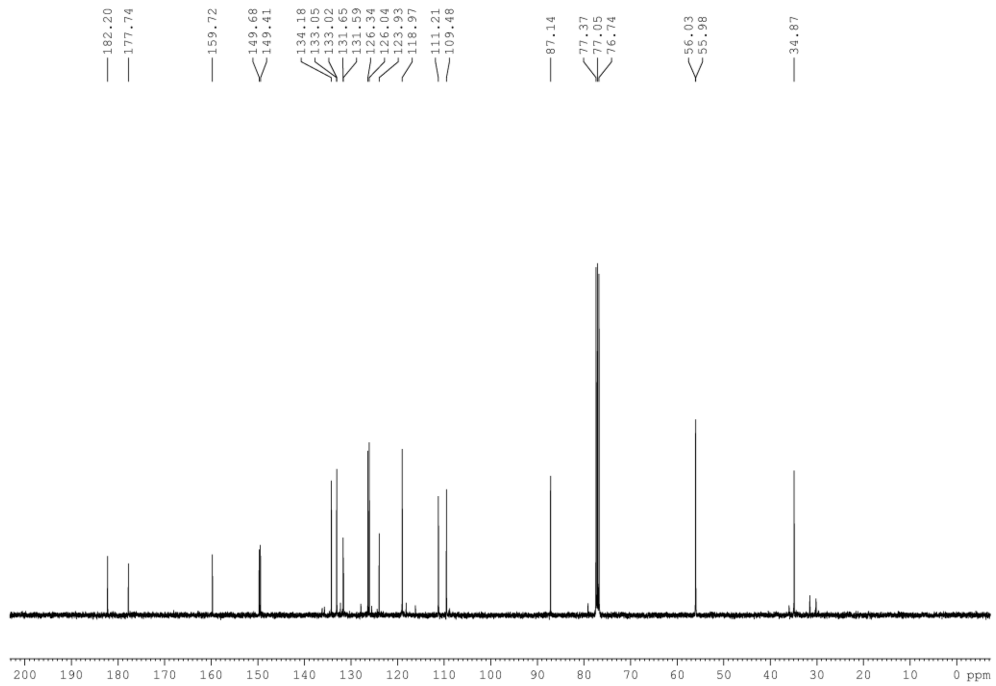

SI-Figure S28. ^{13}C NMR spectrum of compound **5b**.

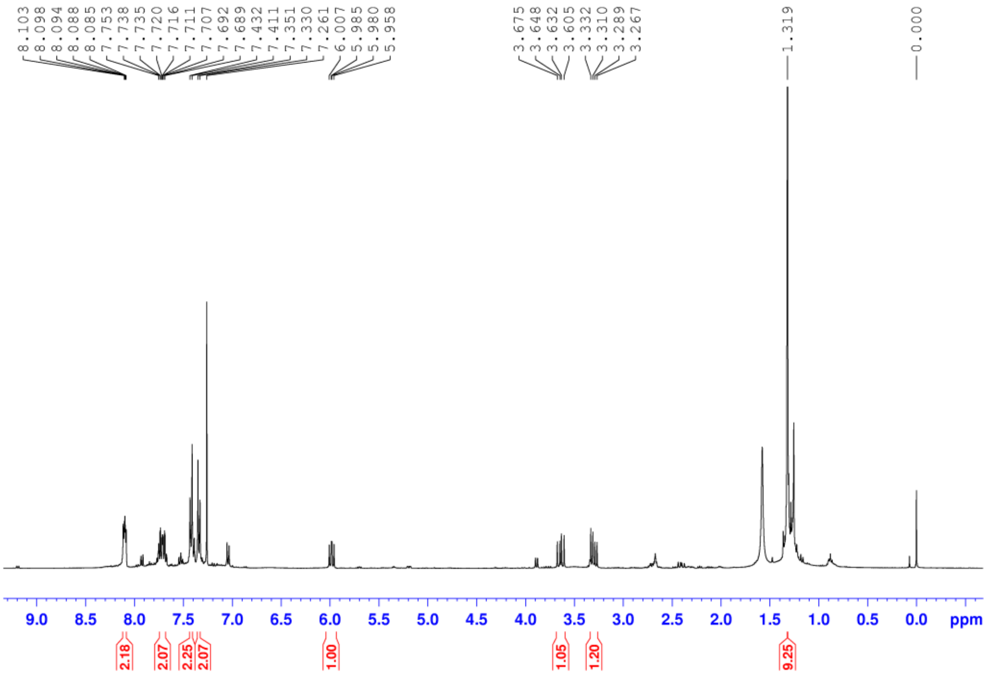

SI-Figure S29. ^{1}H NMR spectrum of compound **5c**.

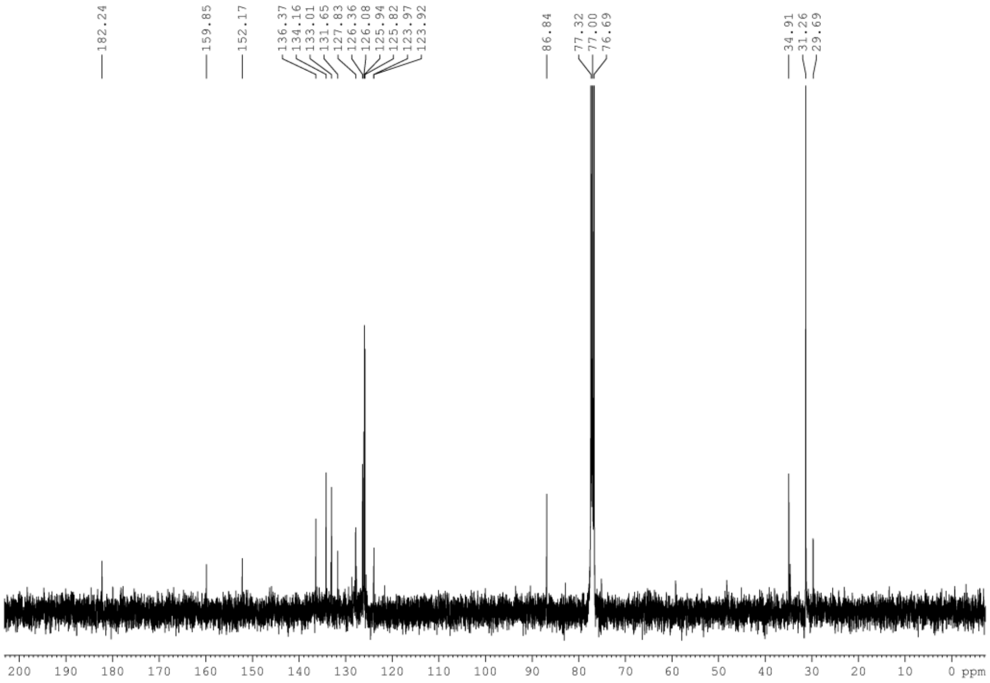

SI-Figure S30. ^{13}C NMR spectrum of compound **5c**.

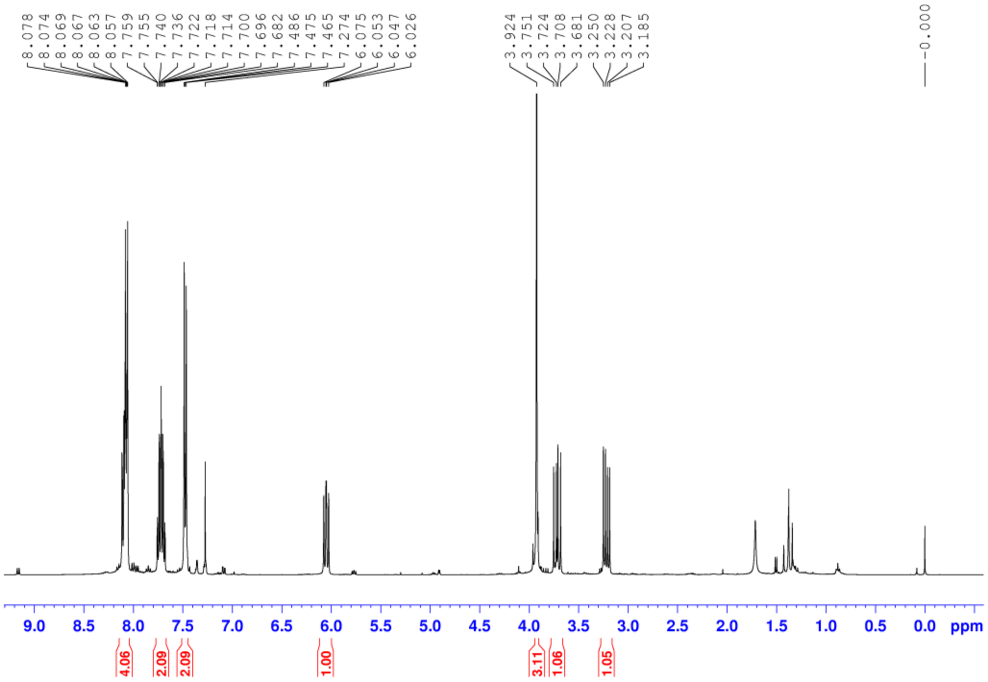

SI-Figure S31. ^{1}H NMR spectrum of compound **5d**.

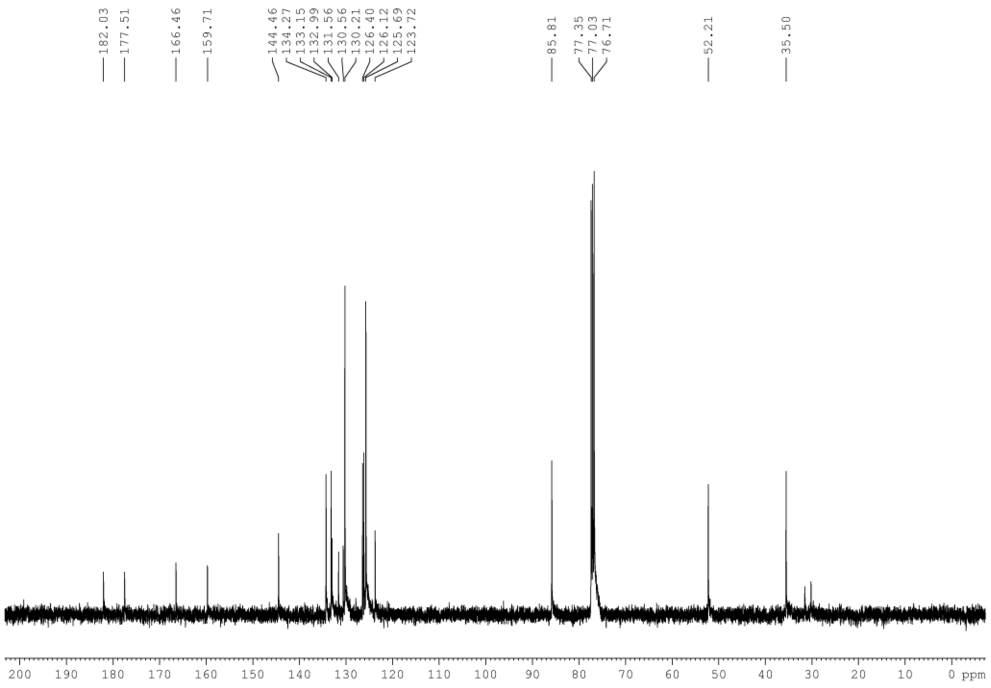

SI-Figure S32. ^{13}C NMR spectrum of compound **5d**.

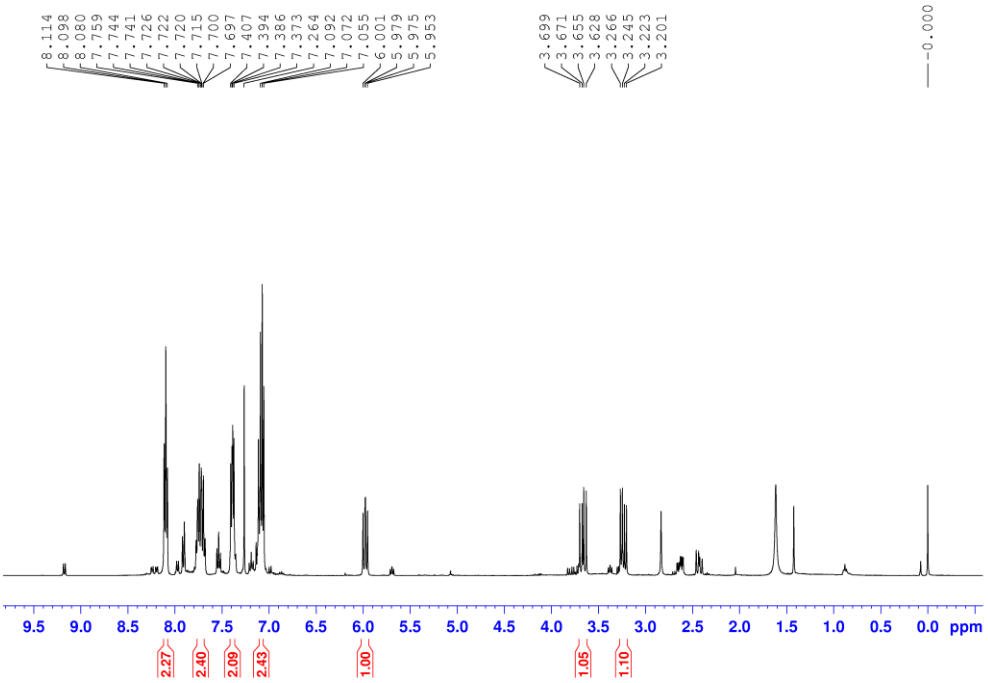

SI-Figure S33. ^{1}H NMR spectrum of compound **5e**.

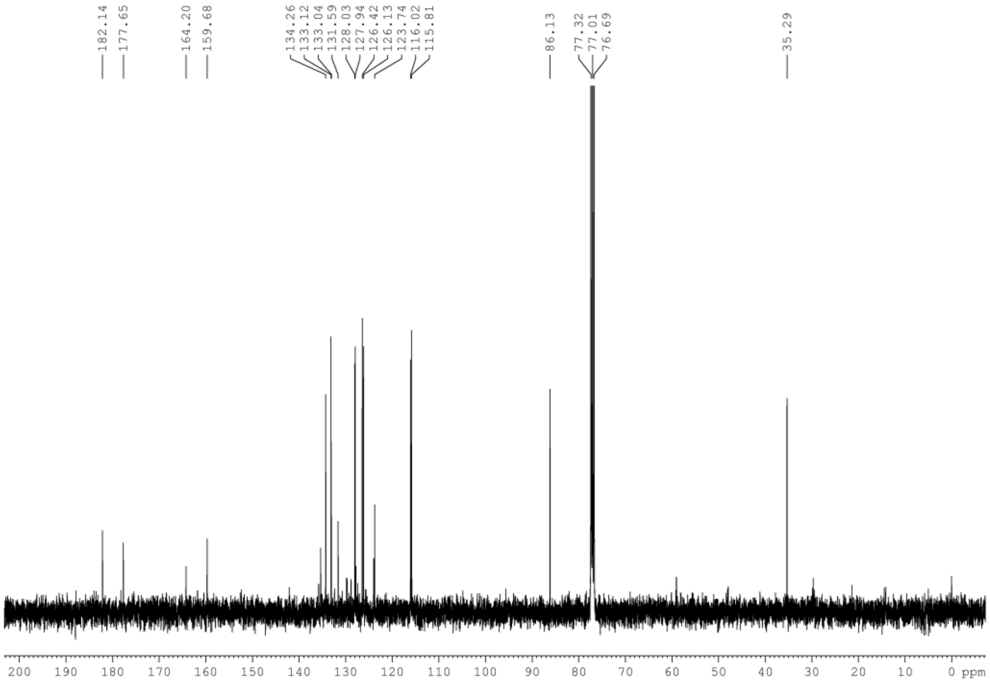

SI-Figure S34. ^{13}C NMR spectrum of compound **5e**.

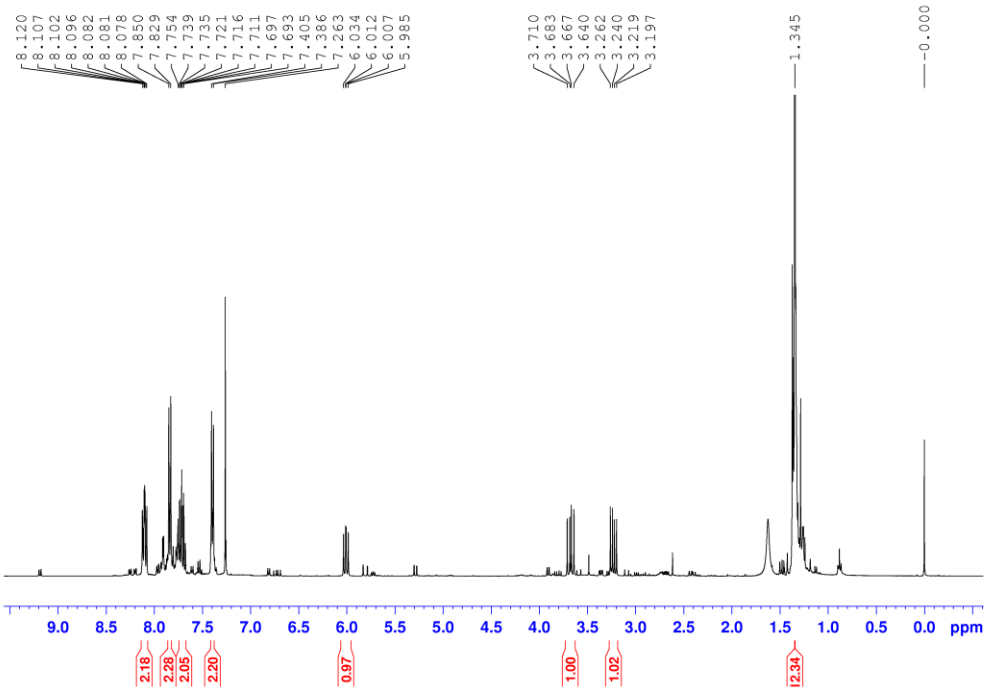

SI-Figure S35. ^{1}H NMR spectrum of compound **5f**.

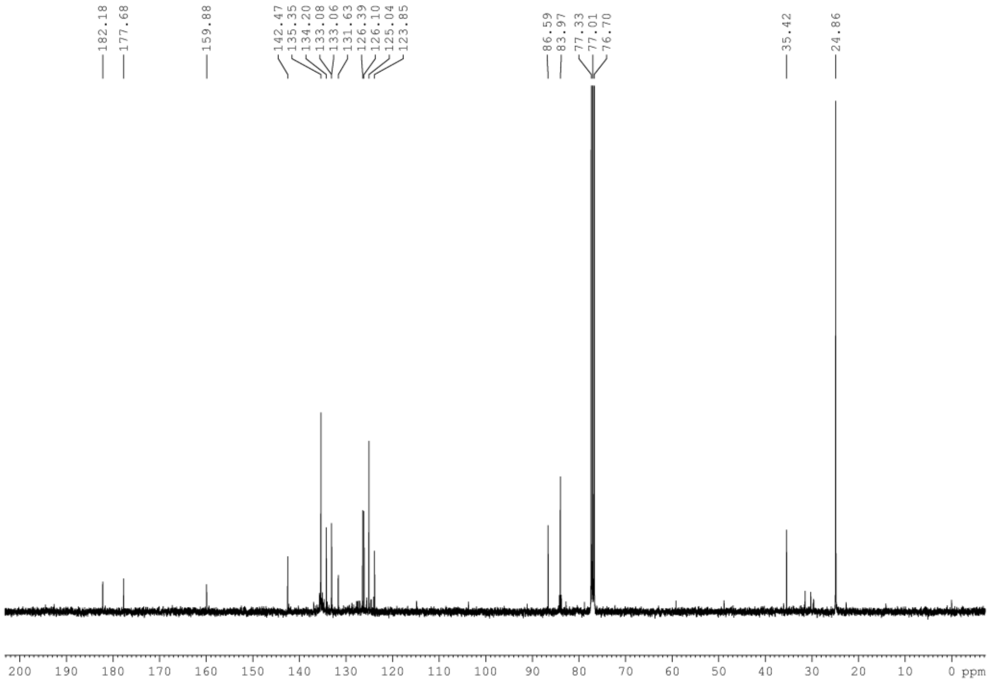

SI-Figure S36. ^{13}C NMR spectrum of compound **5f**.

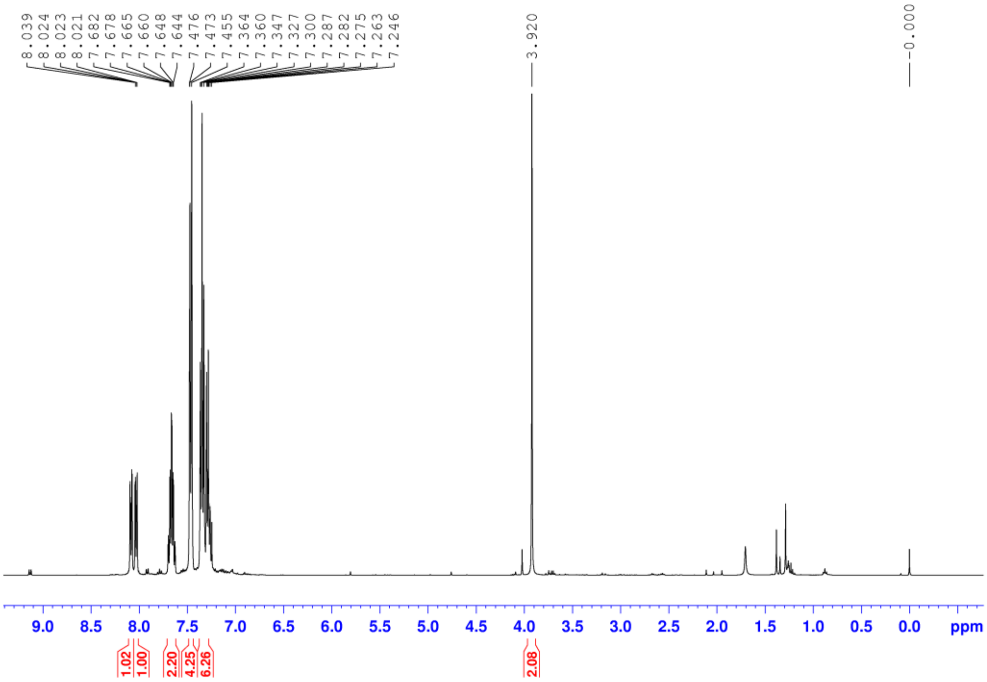

SI-Figure S37. ^{1}H NMR spectrum of compound **5g**.

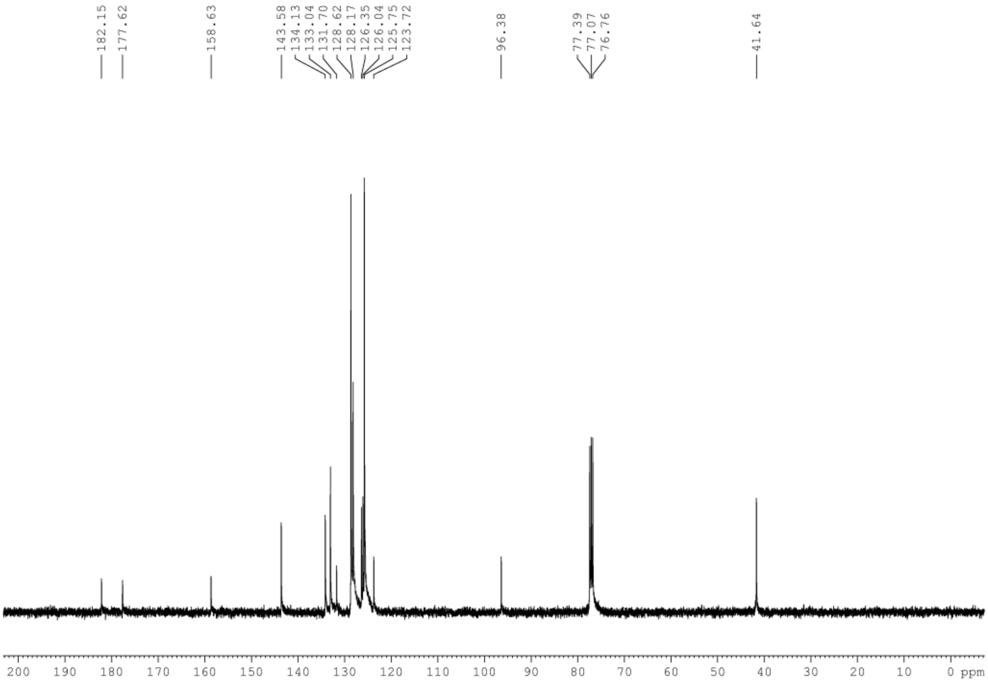

SI-Figure S38. ^{13}C NMR spectrum of compound **5g**.

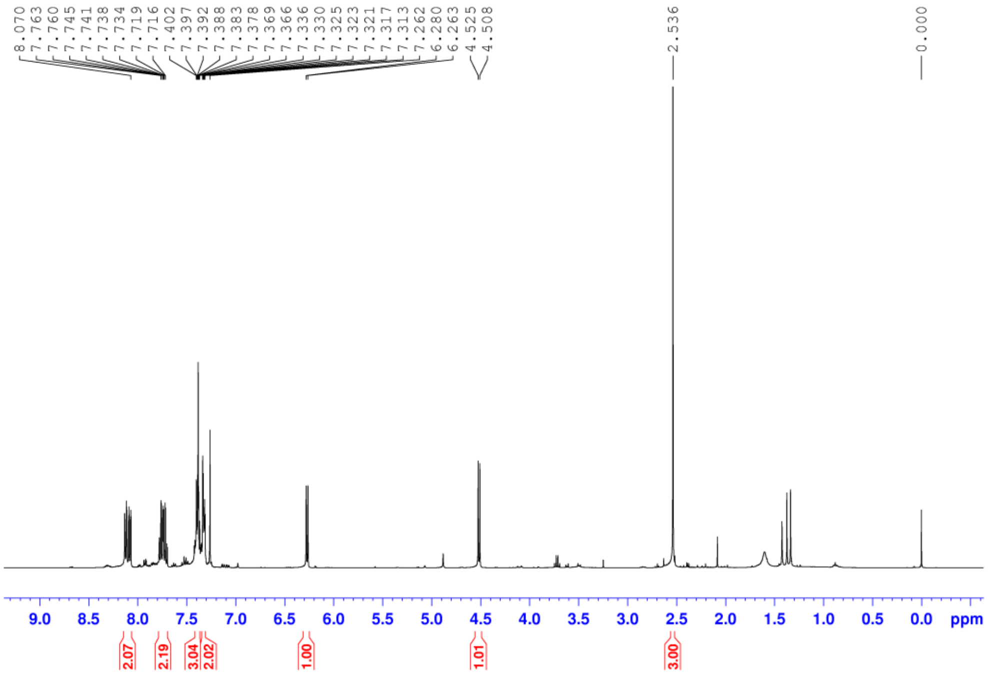

SI-Figure S39. ^{1}H NMR spectrum of compound **5h**.

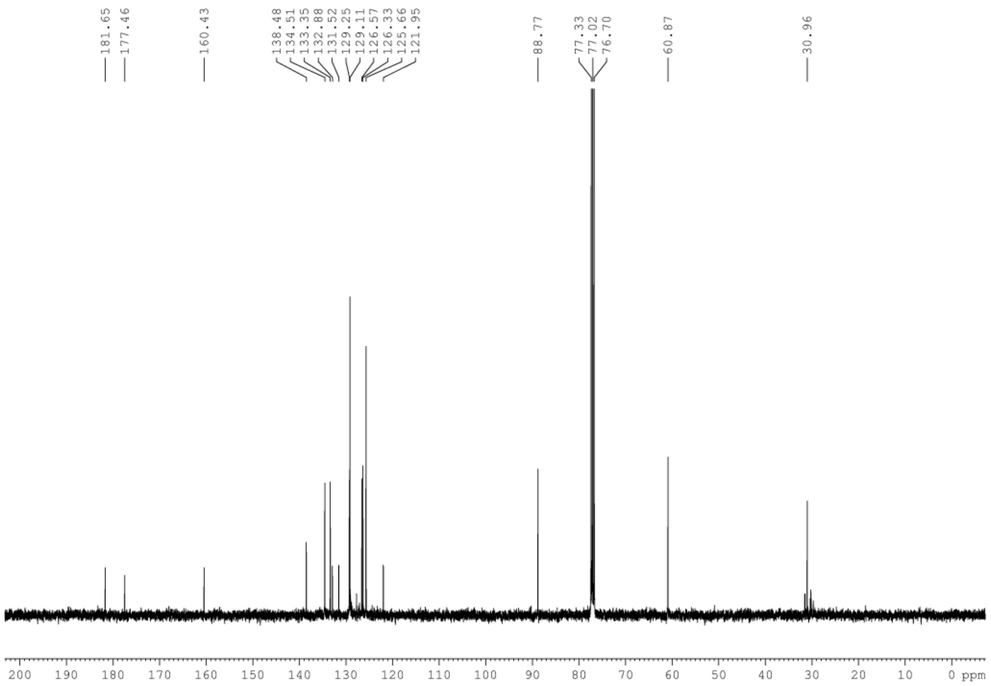

SI-Figure S40. ^{13}C NMR spectrum of compound **5h**.

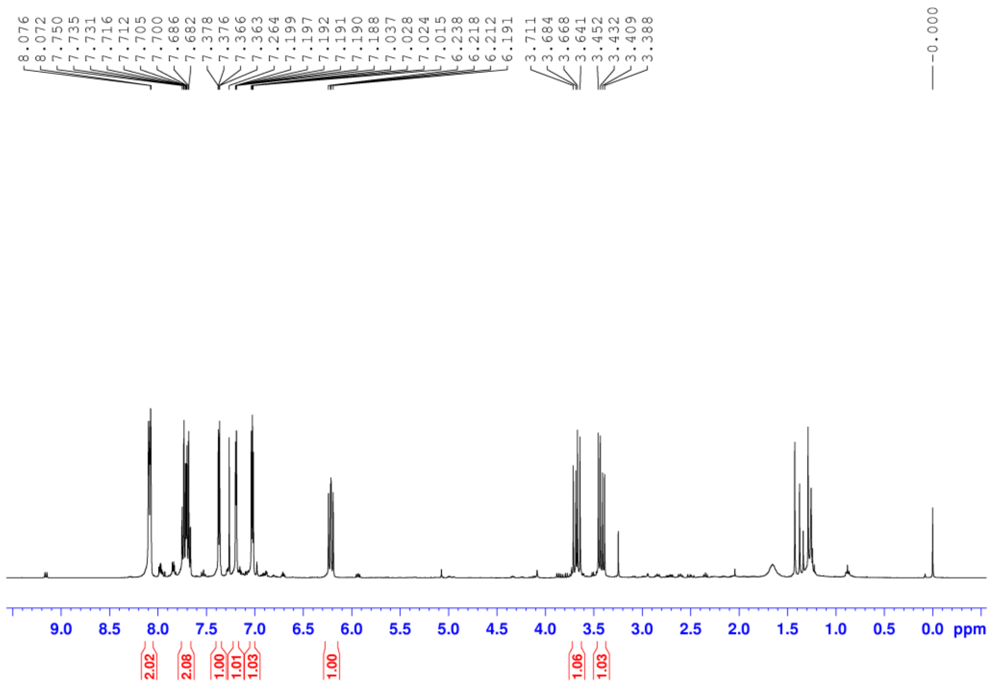

SI-Figure S41. ^{1}H NMR spectrum of compound **5i**.

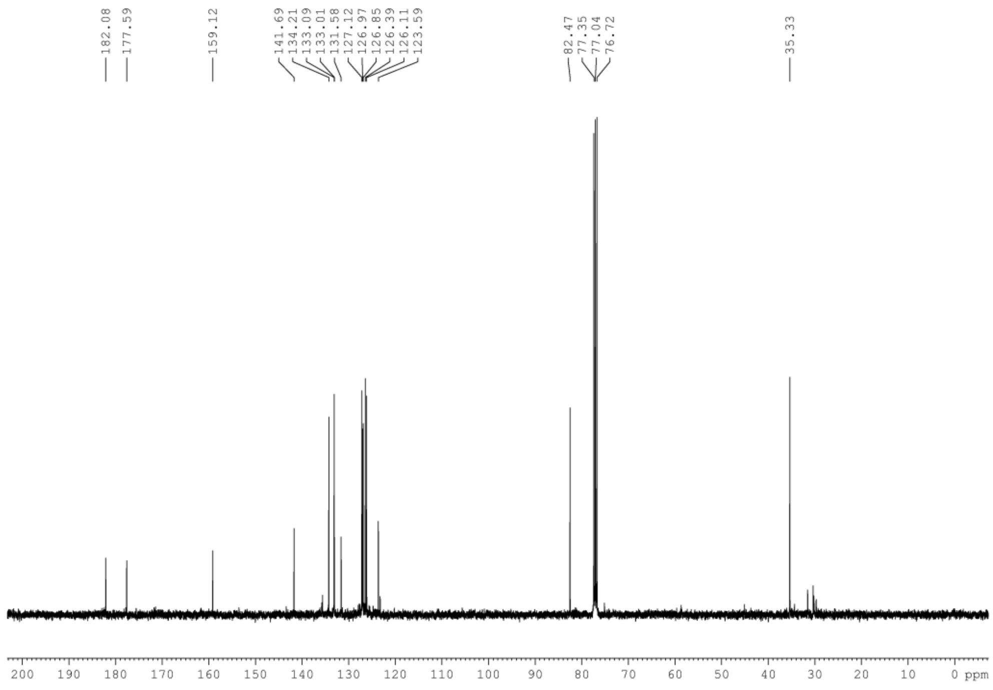

SI-Figure S42. ^{13}C NMR spectrum of compound **5i**.

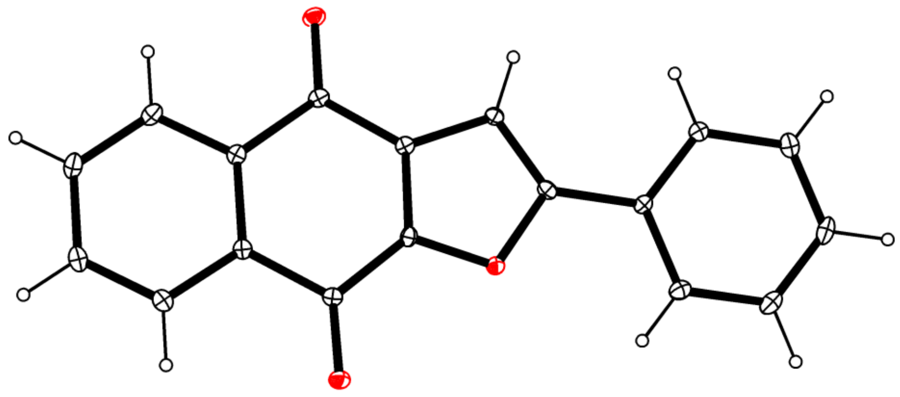

SI-Figure S43. ORTEP diagram of crystal structures of **3a**.

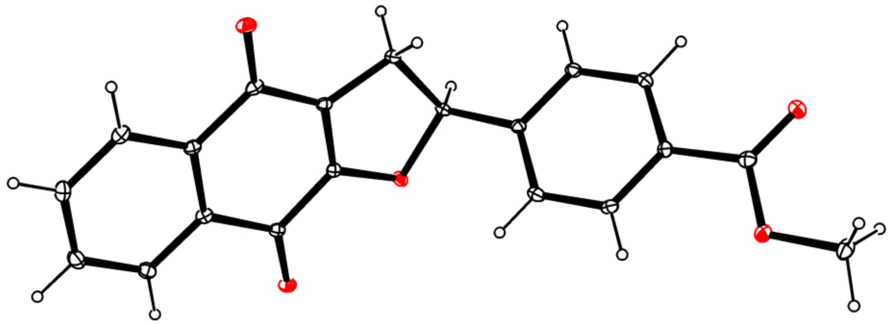

SI-Figure S44. ORTEP diagram of crystal structures of **5d**.
